# Evaluating bias in target trial emulation for heart failure across statistical and deep learning methods

**DOI:** 10.1038/s41467-026-74999-6

**Published:** 2026-07-13

**Authors:** Zhengxian Fan, Qianqian Yang, Yifan Hu, Goodarz Danaei, George Davey Smith, Shishir Rao, Kazem Rahimi

**Affiliations:** 1 Deep Medicine, Nuffield Department of Women’s & Reproductive Health, University of Oxford, Oxford, United Kingdom; 2 Department of Global Health and Population & Department of Epidemiology, Harvard T.H. Chan School of Public Health, Boston, MA USA; 3 MRC Integrative Epidemiology Unit, University of Bristol; Bristol Medical School, University of Bristol; NIHR Bristol Biomedical Research Centre, Bristol, UK

**Keywords:** Epidemiology, Statistical methods, Heart failure

## Abstract

Target trial emulation (TTE) is increasingly used for causal inference from observational data, but remains vulnerable to confounding by indication, and whether advanced adjustment methods mitigate this bias is unclear. Using Clinical Practice Research Datalink Aurum, we emulate target trials of beta-blockers (positive control) and digoxin (negative control) versus usual care on two-year all-cause mortality in patients with heart failure with reduced ejection fraction. We apply four adjustment strategies: propensity score matching, inverse probability of treatment weighting, targeted maximum likelihood estimation, and a Transformer-based deep learning approach. No method reproduces the randomised controlled trial (RCT) benchmarks: all suggest neutral or harmful effects for beta-blockers and elevated mortality for digoxin. In semi-synthetic simulation, all methods recover the true effects when confounders are observed, yet fail in real-world data. TTE, even with advanced adjustment, may not yield trial-equivalent estimates when confounding is strong; randomised evidence remains essential for clinical and policy decisions.

## Abstract (web summary)

Across statistical and deep learning adjustment methods, target trial emulations of heart failure therapies in routine care failed to reproduce randomised trial findings, underscoring the importance of randomised evidence when confounding is strong.

## Introduction

In clinical research, randomised controlled trials (RCTs) are considered the gold standard for evaluating the efficacy and safety of medical interventions. However, the substantial resources, ethical considerations, and extended durations required often make RCTs impractical for assessing every potential effect of interventions across diverse patient populations. Consequently, there is a growing interest in alternative methodologies capable of deriving reliable causal inferences from observational data, despite their inherent susceptibility to various biases^1^.

Target trial emulation (TTE) is an increasingly adopted framework designed to apply the principles of RCTs to observational data. This approach involves specifying a hypothetical trial protocol with eligibility criteria, treatment strategies, outcomes, and follow-up periods. By aligning the start of follow-up (‘time zero’) with the point at which eligibility is met, and treatment assignment is determined, the framework establishes a common baseline across comparison groups and mitigates design-related biases, such as immortal time bias^2^. The UK’s National Institute for Health and Care Excellence (NICE) highlights TTE in its Real-World Evidence Framework, advocating its use to enhance both the rigour and transparency of observational investigations^3^.

Despite growing adoption and regulatory endorsement as a complement to RCTs, the ability of TTE to reproduce RCT findings remains disputable. Studies that have sought to emulate multiple trials have found that only around half of the emulations achieved results concordant with those of the original RCTs, even when studies fully specified the target trial^4,5^. In practice, however, many TTE studies cannot fully avoid the biases inherent in observational analyses. In one systematic review of TTE, 28% of TTE studies were judged to be at serious risk of bias, and none were classified as having a low risk of bias due to confounding^6^. This may itself be an underestimate, as publication bias may favour the reporting of concordant benchmarking studies once the corresponding RCT findings are known. Prospective TTE evaluations undertaken before publication of the corresponding RCT are uncommon, and in a recent prominent case, the later RCT findings differed from those of the earlier TTE report^7,8^.

Cognisant of the persistent risk of confounding in observational analyses conducted within the TTE framework, researchers have adopted a range of methods for causal inference^6^. Common approaches to adjust for confounding include propensity score matching (PSM) and inverse probability of treatment weighting (IPTW). While simple and widely applied, these approaches may be insufficient in the presence of complex confounding structures^9^. More advanced analytical techniques, including “doubly robust” estimators such as targeted maximum likelihood estimation (TMLE) and emerging deep learning-based (DL) causal inference models, have the potential to yield less biased estimates, particularly in the presence of confounding by indication when rich longitudinal data are available^10,11^. However, a systematic evaluation of such approaches within the TTE framework has not been reported.

In this study, we addressed this gap through a detailed evaluation of the TTE methodology in the context of heart failure (HF), a complex clinical condition marked by considerable heterogeneity in comorbidities, clinical presentations, and disease trajectories, making it particularly challenging to obtain unbiased treatment effect estimates from observational data^12^. To evaluate the reliability of the TTE framework, we utilised positive and negative controls to benchmark its ability to replicate established RCT outcomes in HF. Hypothesising that more complex adjustment methods are more likely to mirror RCT results, we employed a range of adjustment methods, including traditional statistical techniques, such as PSM, IPTW, and TMLE, which depend on manual covariate specification, alongside emerging DL approaches that leverage high-dimensional electronic health record (EHR) data without pre-specification of covariates^11^.

## Results

This study used the TTE framework with observational data to estimate the effects of beta-blockers and digoxin, compared with usual care, on all-cause mortality in patients with heart failure with reduced ejection fraction (HFrEF). RCTs have shown that beta-blockers improve survival in HFrEF^13^, making them a suitable positive control. By contrast, the DIG trial, the only major RCT of digoxin in HFrEF, showed no reduction in mortality^14^, making digoxin a suitable negative control.

**Fig. 1:**
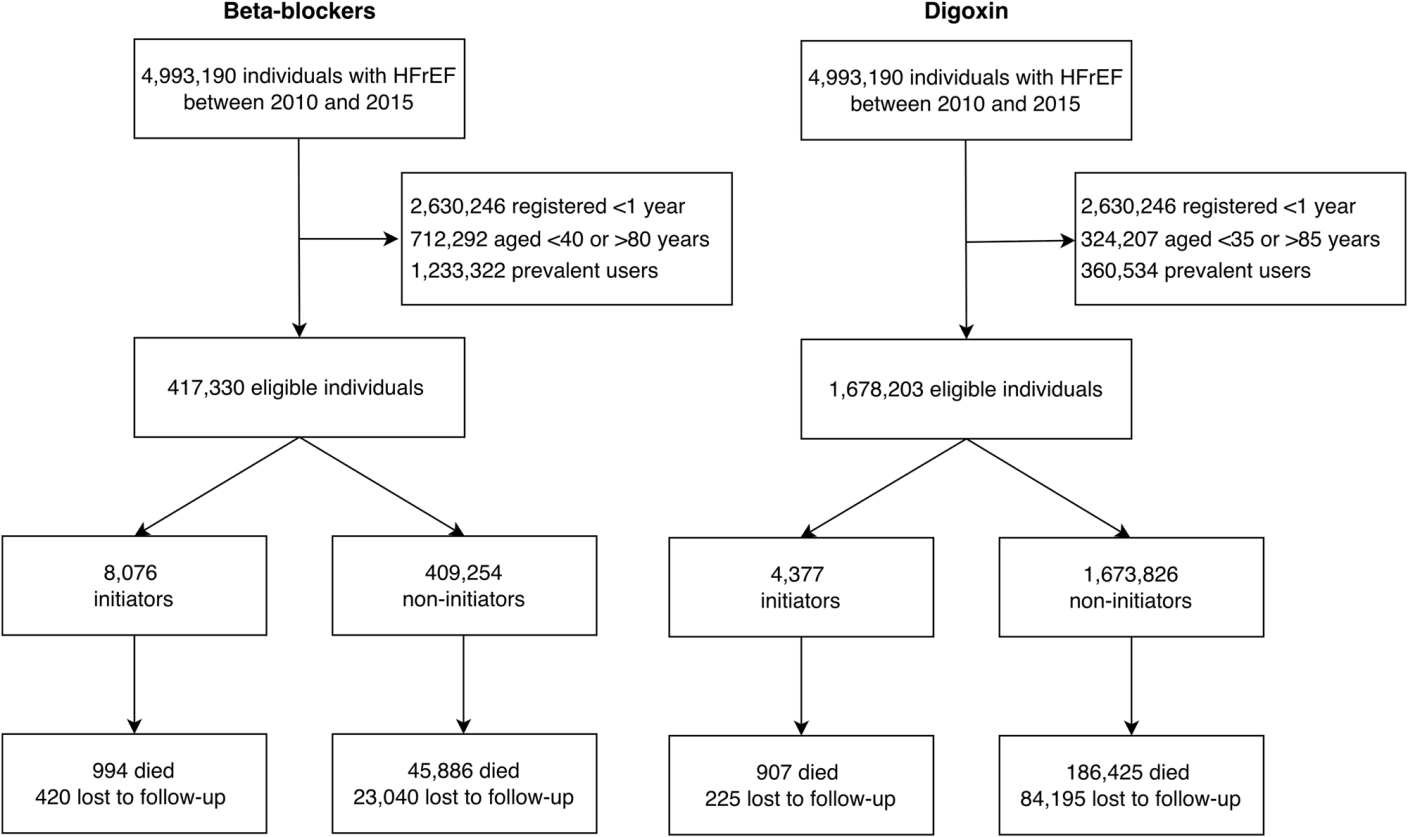
Selection of eligible individuals for target trial emulations of beta-blocker and digoxin therapy. HFrEF, heart failure with reduced ejection fraction.

Using primary care records from the Clinical Practice Research Datalink (CPRD) Aurum linked to hospital admissions and mortality data, we emulated a sequence of monthly target trials of beta-blockers or digoxin initiation versus usual care among adults with HFrEF between January 1, 2010, and January 1, 2015. Cohort selection is summarised in Fig. 1. In total, 417,330 eligible individuals contributed to the beta-blocker trials (8076 initiators; 409,254 non-initiators) and 1,678,203 to the digoxin trials (4377 initiators; 1,673,826 non-initiators). Target trial specifications and emulation details are provided in Supplementary Tables 1, 2 (see also “Methods”). Median follow-up was 2.0 years (interquartile range (IQR), 2.0–2.0) across both cohorts. Treatment adherence, assessed using proportion of days covered (PDC), was high for beta-blockers (median 85.5%, IQR 55.9–93.7%) and digoxin (median 83.0%, IQR 45.8–93.7%).

### Baseline characteristics

Baseline characteristics at trial entry are summarised in Table 1. In the beta-blockers cohort, initiators were slightly younger than non-initiators (mean age 66.4 vs 68.1 years). Pulmonary comorbidity was less common among initiators, including asthma (23.6% vs 34.4%) and chronic obstructive pulmonary disease (COPD; 21.4% vs 28.1%). Initiators had modestly higher prevalence of atrial fibrillation (AF; 40.5% vs 37.3%) and valve disorders (32.9% vs 25.9%). Differences in background pharmacotherapy were more pronounced: initiators more frequently received angiotensin-converting enzyme inhibitors (ACEi; 73.4% vs 56.4%), aldosterone antagonists (28.2% vs 14.3%) and diuretics (67.3% vs 52.3%).

**Table 1:** Baseline characteristics of emulated trial cohorts

Characteristic	Beta-blockers		Digoxin	
	Initiators (*N* = 8076)	Non-initiators (*N* = 409,254)	Initiators (*N* = 4377)	Non-initiators (*N* = 1,673,826)
Age (years, SD)	66.4 (10.3)	68.1 (9.6)	71.0 (10.7)	69.4 (11.3)
**Demographics** ***n* (%)**				
**Sex**				
Male	5591 (69.2%)	274,925 (67.2%)	3012 (68.8%)	1,164,015 (69.5%)
Female	2485 (30.8%)	134,329 (32.8%)	1365 (31.2%)	509,811 (30.5%)
**Ethnicity** ***n* (%)**				
White	7388 (91.5%)	378,448 (92.5%)	4147 (94.7%)	1,541,921 (92.1%)
South Asian	268 (3.3%)	13,725 (3.4%)	102 (2.3%)	63,019 (3.8%)
Black	278 (3.4%)	10,543 (2.6%)	81 (1.9%)	42,006 (2.5%)
Other	107 (1.3%)	4666 (1.1%)	43 (1.0%)	20,983 (1.3%)
Unknown	35 (0.4%)	1872 (0.5%)	4 (0.1%)	5897 (0.4%)
**Index of multiple deprivation** ***n* (%)**				
Quintile 1 (least deprived)	1458 (18.1%)	77,613 (19.0%)	859 (19.6%)	325,939 (19.5%)
Quintile 2	1516 (18.8%)	79,853 (19.5%)	885 (20.2%)	330,872 (19.8%)
Quintile 3	1594 (19.7%)	81,701 (20.0%)	896 (20.5%)	326,788 (19.5%)
Quintile 4	1680 (20.8%)	80,133 (19.6%)	862 (19.7%)	332,596 (19.9%)
Quintile 5 (most deprived)	1818 (22.5%)	89,438 (21.9%)	868 (19.8%)	356,365 (21.3%)
**Smoking status** ***n* (%)**				
Ex-Smoker	2758 (34.2%)	138,436 (33.8%)	1613 (36.9%)	594,904 (35.5%)
Non-Smoker	2290 (28.4%)	113,898 (27.8%)	1207 (27.6%)	487,080 (29.1%)
Smoker	1954 (24.2%)	90,736 (22.2%)	895 (20.4%)	352,349 (21.1%)
**Clinical measurements (Mean, SD)**				
SBP (mmHg)	128.8 (19.4)	129.5 (16.4)	120.9 (18.9)	127.3 (16.9)
BMI (kg/m²)	29.4 (6.5)	29.5 (6.5)	29.7 (6.6)	29.5 (6.1)
HDL-C (mmol/L)	1.3 (0.4)	1.4 (0.4)	1.3 (0.4)	1.3 (0.4)
TC (mmol/L)	4.6 (1.2)	4.5 (1.1)	4.2 (1.1)	4.3 (1.1)
eGFR (mL/min/1.73 m²)	73.2 (22.2)	72.5 (21.4)	64.7 (21.8)	69.0 (22.2)
Heart Rate (bpm)	79.6 (16.9)	76.5 (13.9)	82.7 (20.6)	71.4 (13.1)
**Comorbidities** ***n* (%)**				
Asthma	1906 (23.6%)	140,735 (34.4%)	1048 (23.9%)	337,306 (20.2%)
Atrial fibrillation	3273 (40.5%)	152,468 (37.3%)	3805 (86.9%)	575,994 (34.4%)
Chronic kidney disease	2386 (29.5%)	131,611 (32.2%)	1906 (43.5%)	635,511 (38.0%)
COPD	1732 (21.4%)	115,057 (28.1%)	1063 (24.3%)	296,112 (17.7%)
Diabetes	2289 (28.3%)	116,733 (28.5%)	1350 (30.8%)	497,164 (29.7%)
Dyslipidaemia	2528 (31.3%)	135,922 (33.2%)	1710 (39.1%)	667,614 (39.9%)
Haematological cancer	232 (2.9%)	10,086 (2.5%)	166 (3.8%)	43,731 (2.6%)
Hypertension	5181 (64.2%)	280,480 (68.5%)	3280 (74.9%)	1,214,739 (72.6%)
Ischaemic heart disease	4718 (58.4%)	231,365 (56.5%)	2666 (60.9%)	1,150,936 (68.8%)
Obesity	1452 (18.0%)	73,390 (17.9%)	831 (19.0%)	285,080 (17.0%)
Peripheral artery disease	1007 (12.5%)	54,662 (13.4%)	634 (14.5%)	219,555 (13.1%)
Pulmonary embolism	226 (2.8%)	12,171 (3.0%)	173 (4.0%)	42,962 (2.6%)
Solid cancer	1031 (12.8%)	56,486 (13.8%)	654 (14.9%)	220,898 (13.2%)
Stroke	995 (12.3%)	59,782 (14.6%)	732 (16.7%)	231,145 (13.8%)
Thyroid disorders	1096 (13.6%)	60,511 (14.8%)	716 (16.4%)	241,488 (14.4%)
Valve disorders	2658 (32.9%)	105,874 (25.9%)	1779 (40.6%)	453,070 (27.1%)
**Baseline medications** ***n* (%)**				
ACEi	5925 (73.4%)	230,752 (56.4%)	3139 (71.7%)	1,117,332 (66.8%)
ARB	1597 (19.8%)	92,201 (22.5%)	1052 (24.0%)	397,451 (23.7%)
Aldosterone	2275 (28.2%)	58,701 (14.3%)	1889 (43.2%)	396,801 (23.7%)
Anticoagulant	2486 (30.8%)	111,990 (27.4%)	2888 (66.0%)	434,537 (26.0%)
Antiplatelet	4671 (57.8%)	192,289 (47.0%)	2356 (53.8%)	1,034,261 (61.8%)
CCB	2177 (27.0%)	124,287 (30.4%)	1123 (25.7%)	371,656 (22.2%)
Beta-blockers	—	—	3588 (82.0%)	1,222,184 (73.0%)
Digoxin	1277 (15.8%)	61,153 (14.9%)	—	—
Diuretic	5438 (67.3%)	213,920 (52.3%)	3756 (85.8%)	1,024,593 (61.2%)
Statin	5205 (64.5%)	256,946 (62.8%)	2841 (64.9%)	1,234,811 (73.8%)
Vasodilator	814 (10.1%)	42,738 (10.4%)	663 (15.1%)	240,148 (14.3%)

*ACEi,* angiotensin-converting enzyme inhibitor; *ARB*, angiotensin II receptor blocker; *BMI*, body mass index; *CCB*, calcium channel blocker; *COPD*, chronic obstructive pulmonary disease; *eGFR*, estimated glomerular filtration rate; *HDL-C*, high-density lipoprotein cholesterol; *IMD*, index of multiple deprivation; *SBP*, systolic blood pressure; *SD*, standard deviation; *TC*, total cholesterol. **Missingness (%):** Beta-blockers cohort: Heart Rate 58.0, BMI 26.8, HDL-C 25.6, TC 17.9, Smoking status 16.1, eGFR 13.6, SBP 7.1, IMD 0.1; Digoxin cohort: Heart Rate 51.9, BMI 25.6, HDL-C 21.4, Smoking status 15.3, TC 13.1, eGFR 9.8, SBP 3.3, IMD 0.1.

The digoxin cohort exhibited greater baseline imbalance between initiators and non-initiators. AF was markedly more prevalent among initiators than non-initiators (86.9% vs 34.4%). Initiators also had higher prevalence of chronic kidney disease (43.5% vs 38.0%) and hypertension (74.9% vs 72.6%), and more frequent use of anticoagulants (66.0% vs 26.0%) and diuretics (85.8% vs 61.2%).

### Benchmarking observational estimates against randomised trial results

To estimate the intention-to-treat effect of therapy initiation on 2-year all-cause mortality and benchmark these estimates against established RCT results, we applied several adjustment strategies designed to mitigate confounding. These included two commonly used propensity score-based approaches, PSM and IPTW, with propensity scores estimated from 39 prespecified baseline covariates. This covariate set comprised all variables detailed in Table 1, as well as geographical region and year of the trial enrolment.

We also applied TMLE using the same covariate set; as a doubly robust estimator, TMLE models treatment assignment and outcome jointly and can provide consistent estimates if either the treatment model or the outcome model is correctly specified^15^. In parallel, we evaluated a Transformer-based DL approach that leverages the full pre-baseline longitudinal EHR sequence to learn latent representations of patient trajectories for predicting treatment and outcome^11^. By incorporating high-dimensional longitudinal information beyond manually specified covariates, this approach is intended to account for more complex confounding structures captured in routine care data. The DL estimator was implemented within the same doubly robust estimation framework as TMLE. For weighting-based estimators (IPTW, TMLE and DL), we applied Stürmer trimming at the 1st and 99th percentiles of the propensity score distribution in the main analysis to reduce the influence of extreme weights^16^. Further implementation details are provided in the “Methods”.

**Fig. 2:**
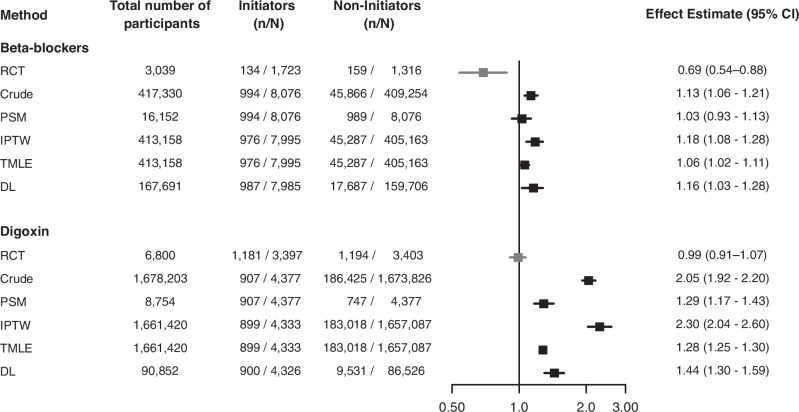
Relative effect estimates of initiating beta-blockers and digoxin on 2-year all-cause mortality in patients with heart failure with reduced ejection fraction (HFrEF). Estimates from crude analyses, propensity score matching (PSM), and inverse probability of treatment weighting (IPTW) are presented as hazard ratios; estimates from targeted maximum likelihood estimation (TMLE) and deep learning (DL) are presented as risk ratios. Squares represent point estimates and error bars represent 95% confidence intervals (CI); the grey square denotes the RCT benchmark. RCT, randomised controlled trial; n, number of deaths; N, group size.

Effect estimates with 95% confidence intervals (CIs) for 2-year all-cause mortality associated with initiation of beta-blockers and digoxin are shown in Fig. 2, with observational estimates from each adjustment method presented alongside the corresponding RCT estimates. For beta-blockers, randomised evidence demonstrated a substantial reduction in mortality compared with placebo (0.69, 95% CI 0.54–0.88). In crude observational analyses, initiation was associated with higher mortality (1.13, 1.06–1.21). While adjustment attenuated this association, none of the approaches successfully replicated the protective effect observed in the RCT. Estimates were closest to the null under PSM (1.03, 0.93–1.13), remained modestly above unity under TMLE (1.06, 1.02–1.11), and were higher under IPTW (1.18, 1.08–1.28) and DL (1.16, 1.03–1.28).

For digoxin, the RCT indicated a neutral effect on mortality compared with placebo (0.99, 0.91–1.07). In contrast, in crude analyses, digoxin initiation was associated with markedly higher mortality compared with non-initiation (2.05, 1.92–2.20). Although adjustment reduced the magnitude of this association for some methods, all adjusted estimates suggested a harmful effect (PSM 1.29, 1.17–1.43; IPTW 2.30, 2.04–2.60; TMLE 1.28, 1.25–1.30; DL 1.44, 1.30–1.59).

Propensity score distributions estimated using logistic regression and the DL model are shown in Supplementary Fig. 1. The digoxin cohort exhibited less overlap between initiators and non-initiators than the beta-blockers cohort, likely reflecting more selective prescribing of digoxin in routine clinical practice. DL-derived propensity scores showed greater separation between treated and untreated individuals than logistic regression-based scores in both cohorts, indicating that the model leveraged additional longitudinal EHR information relevant to treatment assignment beyond the prespecified baseline covariates.

### Sensitivity and subgroup analyses

To evaluate the robustness of our results, we performed sensitivity analyses across varying trimming thresholds. We applied Stürmer trimming for IPTW, TMLE, and DL analyses, varying the threshold from 1% to 10%. Across all thresholds, effect estimates remained stable with only modest changes as the proportion trimmed increased. These results consistently suggest a neutral or modestly elevated mortality risk associated with beta-blocker initiation and harmful effects associated with digoxin initiation (Supplementary Figs. 2, 3).

Because DL models can be sensitive to class imbalance, particularly when non-initiators substantially outnumber initiators^17^, we implemented 1:20 treatment-to-control subsampling in the primary analysis to facilitate model training. We verified that DL estimates were stable across a range of sampling ratios from 1:5 to 1:20 (Supplementary Table 3).

In analyses stratified by baseline AF status, beta-blocker estimates were broadly similar among individuals with and without AF. For digoxin, all adjustment methods showed elevated mortality risk irrespective of AF status; however, the magnitude of association was substantially greater among individuals without AF than among those with AF (Supplementary Fig. 4).

### Semi-synthetic simulation

To complement the empirical benchmarking and examine the impact of unmeasured confounding under controlled conditions, we conducted a semi-synthetic simulation anchored to the observed covariate distribution of the beta-blockers cohort. Treatment assignment and mortality outcomes were generated under a prespecified data-generating mechanism with a known ground-truth relative effect. Five chronic conditions were specified as confounders: hypertension, diabetes, ischaemic heart disease, thyroid disorder and COPD. To represent scenarios in which key clinical confounders are unknown, we evaluated methods under three settings: (1) no omitted confounders (all specified covariates adjusted); (2) one omitted confounder (thyroid disorder excluded from adjustment); and (3) two omitted confounders (thyroid disorder and COPD excluded).

**Fig. 3:**
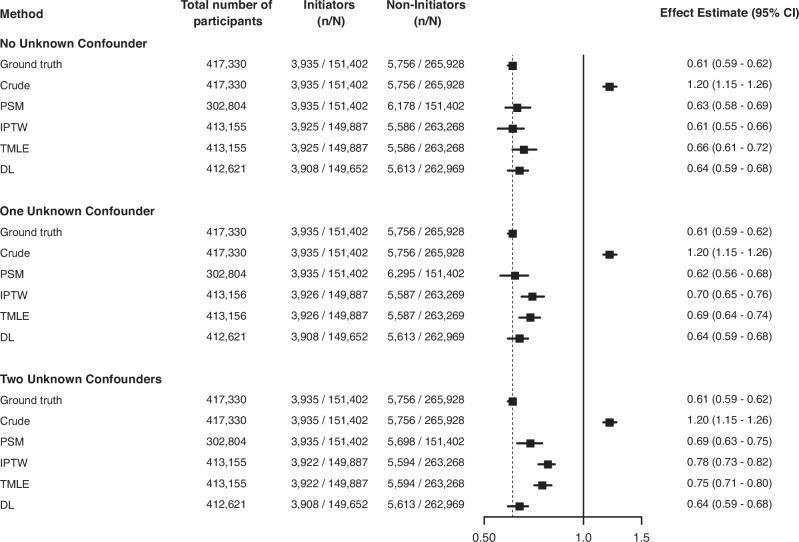
Treatment effect estimates in a simulated cohort with varying levels of unmeasured confounding. Data were generated using a semi-synthetic simulation anchored to the observed baseline covariate distribution of the heart failure population. Both treatment assignment and mortality outcomes were simulated using a prespecified data-generating mechanism to establish a ground truth treatment effect, represented by the vertical dotted line. The three scenarios illustrate the impact of omitting zero, one, or two key confounders from the covariate selection process to evaluate how results change as unmeasured confounding increases. Estimates from crude analyses, propensity score matching (PSM), and inverse probability of treatment weighting (IPTW) are presented as hazard ratios; estimates from targeted maximum likelihood estimation (TMLE) and deep learning (DL) are presented as risk ratios. Squares represent point estimates, and error bars represent 95% confidence intervals (CI). Across all scenarios, the DL model utilised the full longitudinal electronic health record (EHR) representation without manual covariate selection. n, number of deaths; N, group size.

When all specified confounders were adjusted for, the adjusted methods closely reproduced the ground-truth effect (0.61, 95% CI 0.59–0.62). Estimates were 0.61 (95% CI 0.55–0.66) for IPTW, 0.63 (0.58–0.69) for PSM, and 0.66 (0.61–0.72) for TMLE (Fig. 3), in contrast to the substantially biased crude estimate of 1.20 (1.15–1.26). With one omitted confounder, deviation from the ground truth increased for IPTW (0.70, 0.65–0.76) and TMLE (0.69, 0.64–0.74), whereas PSM (0.62, 0.56–0.68) remained closer to the simulated effect. Under two omitted confounders, divergence increased further, with IPTW and TMLE estimates rising to 0.78 (0.73–0.82) and 0.75 (0.71–0.80), respectively.

Because the DL approach leveraged the full pre-baseline EHR sequence rather than relying on manually specified covariates, its estimates were unchanged across the three scenarios (0.64, 0.59–0.68). In the simulation, logistic regression-based propensity scores placed treated individuals predominantly near 1 and controls near 0, with modest overlap between groups. In contrast, DL-derived propensity scores showed greater separation between treated and untreated individuals, producing two peaks near 0 and 1, consistent with the simulated treatment assignment mechanism (Supplementary Fig. 5). This pattern suggests that the DL model captured information related to treatment assignment from the longitudinal EHR without explicit covariate specification.

## Discussion

In this study, we evaluated whether the TTE framework could reproduce trial benchmarks for the effects of beta-blockers and digoxin on all-cause mortality in patients with HFrEF using CPRD Aurum data. We employed four adjustment methods, including PSM, IPTW, TMLE, and DL, to estimate treatment effects, yet none of the methods reproduced the trial benchmarks. For beta-blockers, none of the approaches replicated either the magnitude or direction of the established survival benefit, instead yielding neutral or harmful estimates. For digoxin, all methods produced estimates consistent with increased mortality, in contrast to the neutral mortality result observed in the trial benchmark. In our semi-synthetic simulation, all methods closely approximated the ground-truth effect when confounders were fully observed and available for adjustment. Notably, the DL model leveraged longitudinal EHR sequences to recover the simulated effect without explicit covariate specification. However, the consistent failure of these advanced methods in our real-world emulation underscores the intractable nature of confounding by indication in routine clinical records for HF populations.

Our findings add to a growing body of work evaluating the generalisability of TTE. The framework has previously been shown to replicate certain RCT results. For instance, it was first applied to demonstrate the protective effects of statins in preventing coronary heart disease^18^, and subsequent applications reproduced RCT findings in contexts such as paclitaxel chemotherapy for breast cancer^19^, statins for cancer prevention^20^, and timing of dialysis initiation in chronic kidney disease^21^. However, these early successes each focused on a single treatment-outcome scenario, leaving it unclear whether they reflect true generalisability or simply selective reporting. When multiple trials are emulated, agreement is mixed. In the RCT-DUPLICATE Initiative, which emulated a mix of active-comparator and placebo-controlled trials, only 6 of 10 emulations aligned with RCT findings despite rigorous efforts to mirror trial design^4^. A subsequent analysis of 32 emulated trials, which used active-comparator new-user designs with propensity score matching throughout, reinforced this variability, with only 56% of the emulations concordant with the original RCTs^5^. Both efforts relied on PSM as the sole method for confounding adjustment. In our study, even after incorporating “doubly robust” methods like TMLE and DL^22^, we still could not fully replicate RCT estimates. This suggests that the challenge lies not just in the choice of adjustment method, but in the underlying biases inherent in observational settings.

A key contribution of this study is the systematic evaluation of treatment effect estimates produced by both traditional statistical methods and DL approaches within the TTE framework, as applied to a complex, multimorbid HF population. By leveraging routine clinical data, we provide empirical insights into the challenges of estimating treatment effects in HF populations. Despite regulatory and methodological endorsements of TTE as a path to RCT-equivalent estimates^3,23^, our findings underscore the ongoing challenges in adequately addressing confounding by indication within the TTE framework. This pattern remained evident even when ‘doubly robust’ adjustment methods such as TMLE and DL were used. The inability to reproduce the neutral mortality signal for digoxin is particularly instructive when viewed alongside contemporary clinical evidence. Digoxin was selected as a negative control on the basis of prior randomised trial evidence indicating no effect on all-cause mortality. More recently, the DIGIT-HF trial evaluated digitoxin, a closely related cardiac glycoside, compared with placebo. The trial reported a reduction in the composite endpoint of death or first hospitalisation for worsening HF, but no clear reduction in all-cause mortality (HR 0.86, 95% CI 0.69–1.07)^24^. Taken together, these findings suggest that cardiac glycosides may reduce HF morbidity without clear evidence of harm with respect to mortality. In contrast, our observational analyses consistently yielded elevated mortality estimates for digoxin. The divergence between contemporary trial findings and routine-care estimates reinforces the importance of randomised evidence in this setting and highlights that, even with advanced adjustment methods and comprehensive longitudinal data, deriving reliable treatment effect estimates remains challenging in complex HF populations.

Our simulation results demonstrate that when all relevant confounders are fully observed or reliably inferable, data-driven models can closely approximate the effects seen in randomised trials without manual covariate specification. When applied to real-world EHR data, however, the same approaches did not reproduce the trial benchmarks. This discordance suggests that in complex clinical settings like HF, strong indication biases persist, and important confounders remain unrecorded or not inferable from routine EHR data^25^, limiting the ability of current methods to generate reliable estimates even under the TTE framework. These issues are especially pronounced when comparing initiators with non-initiators; active-comparator designs may help alleviate some of these imbalances. Although TTE can improve transparency and reduce design-related and selection biases in observational research, it does not by itself resolve confounding by indication. The practical importance of this challenge may be under-appreciated in current emulation studies, especially when routine data do not fully capture the clinical factors underlying treatment decisions. Consequently, even carefully implemented TTEs may struggle when confounding by indication is strong or cannot be adequately addressed^26^.

This study has several limitations. First, although CPRD Aurum is a representative cohort covering approximately 20% of the UK population, most patients lack recorded ejection fraction data. We therefore identified HFrEF mainly through diagnostic codes explicitly indicating reduced ejection fraction, which capture only around 10% of the HF population^27^. Second, although CPRD contains rich clinical data, some structured indicators of HF severity, such as the New York Heart Association (NYHA) functional classification, are missing for over 95% of patients and were therefore not included as covariates. However, HF severity is likely to be proxied through other routinely documented factors in the EHR^28^. Third, we benchmarked against placebo-controlled trials only. Such emulations are especially vulnerable to confounding by indication and may be less robust than active-comparator designs, which compare patients initiating alternative therapies at a more similar point in the clinical decision process^29^. Fourth, we did not conduct extensive subgroup analyses because the primary emulations were not consistent with the benchmark RCT findings, which limited the value of further subgroup analyses. Finally, the estimands differ across methods. PSM restricts the analysis to treated patients and their matched control patients, IPTW and TMLE reweight individuals (with trimming), and the deep learning approach uses subsampling. Consequently, the effect estimates from these approaches are not directly comparable, as each method effectively targets a slightly different population. Differences in effect estimates across methods should therefore not be interpreted as differences in methodological performance, but may in part reflect variation in the underlying target population and potential effect heterogeneity.

In this large evaluation using routine care data, we demonstrate that confounding by indication remains a formidable obstacle in observational research, and that adopting the TTE framework, even when paired with advanced adjustment methods, does not ensure reliable reproduction of RCT results. TTE should be viewed as one useful framework, not a universal solution. Where clinical stakes are high, decisions should prioritise randomised evidence; where trials are infeasible, triangulation across multiple observational designs, the use of negative and positive controls, and extensive, transparent sensitivity analyses are essential for credible inference.

## Methods

### Data sources

This research complies with all relevant ethical regulations. The observational data used in this study were sourced from the CPRD Aurum, a primary care database covering approximately 20% of the UK population^30^. CPRD contains comprehensive data on demographics, diagnoses, prescriptions, laboratory results, and lifestyle factors. It is linked to Hospital Episode Statistics (HES) and the Office for National Statistics (ONS), which provide secondary-care and mortality records, enhancing outcome ascertainment and cohort completeness^31^. The CPRD has ethical approval from the National Research Ethics Service Committee (research ethics committee reference 21/EM/0265) for observational research using anonymised CPRD data. This research was conducted using the CPRD Aurum resource under protocol number 20_095, approved by the CPRD Independent Scientific Advisory Committee. Data use complied with all requirements of the data provider. Informed consent was not required, as CPRD provides de-identified, routinely collected data approved for research use without individual consent.

### Target trial emulation protocol

TTE involves structuring observational data to mirror the design principles of RCTs. This approach identifies eligible individuals within observational datasets and assigns them to treatment strategies according to observed initiation status at baseline. These individuals were then followed from the point of assignment (“time zero”) until the occurrence of the outcome or the end of the specified follow-up period.

In this study, we specified the TTE protocol in accordance with the TARGET guideline^32^. We emulated a series of monthly hypothetical trials for beta-blockers and digoxin. A new round of participant recruitment was initiated at the beginning of each month over a 60-month period, from January 1, 2010, to January 1, 2015. Each monthly enrolment period was treated as an independent trial, and participants were assigned to treatment strategies based on their treatment initiation status during that specific month. We summarised the key components of the target-trial protocol (Supplementary Tables 1, 2) and described them in detail below.

#### Eligibility criteria

Participants were eligible if they had a recorded diagnosis of HFrEF in primary or secondary care before enrolment, identified through clinical codes or a most recent recorded left ventricular ejection fraction (LVEF) of <40%. The code list for identifying HFrEF was sourced from a population-based study of HF subtypes conducted within the CPRD^27^.

To ensure adequate ascertainment of baseline covariates and to implement a one-year washout period for prior treatment exposure, we required at least one year of continuous registration with a general practice before enrolment. Individuals were excluded if they had received a prescription for the target drug during the 12 months preceding enrolment. To align the emulated cohorts with the selection criteria and age distribution of the corresponding benchmark RCTs^13,14^, age requirements were specified separately for each treatment. For the beta-blocker emulation, we restricted eligibility to individuals aged 40–80 years to match the benchmark trial criteria. For the digoxin emulation, although the original DIG trial enrolled adults aged 21 years and older, only a small proportion of participants were older than 80 years^33^. Because routine care includes a greater proportion of very elderly patients with HF than the trial population, directly applying the original age criterion would have produced a less comparable age distribution. We therefore applied an age restriction of 35–85 years for the digoxin emulation. Individuals could contribute to multiple trials if they met eligibility criteria at more than one enrolment period^18,20^. No statistical method was used to predetermine sample size; all individuals meeting the eligibility criteria during the enrolment window were included.

#### Treatment strategy and assignment procedures

Eligible participants were classified into either (1) the treatment group, comprising patients who initiated the target drug within the enrolment month, or (2) the control group, comprising patients who did not initiate the target drug during that period and therefore continued under usual care. In this study, usual care referred to routine clinical management provided through UK primary care. As all participants were actively registered with a general practice, those in the control group were assumed to continue receiving ongoing management for HFrEF through primary care services.

#### Outcomes

The outcome of interest was all-cause mortality, determined through ONS mortality records.

#### Follow-up period

Follow-up began at enrolment and ended at the earliest occurrence of mortality, loss to follow-up, or completion of the 2-year follow-up period. Adherence to treatment was quantified as the PDC during follow-up. The numerator for the PDC was the total number of unique days a patient was supplied with the medication. This was determined from prescription records, with each prescription providing a duration. The denominator was the total number of days in each patient’s follow-up period.

#### Causal contrasts

Our causal contrast of interest was the intention-to-treat effect of initiating beta-blockers (or digoxin) versus usual care at baseline.

#### Identifying assumptions

We assumed conditional exchangeability, meaning that, after adjusting for measured covariates, the groups initiating treatment and not initiating would have had the same risk of mortality if they had followed the same treatment strategy. We also assumed no measurement error in baseline covariates and that dispensing/prescription records served as a reasonable proxy for actual medication use for descriptive adherence summaries.

#### Data analysis plan

We performed intention-to-treat analyses using statistical and deep learning-based adjustment methods to estimate the effects of beta-blockers and digoxin on all-cause mortality.

#### Covariate selection and missing data

For statistical approaches, confounding adjustment required pre-specification of baseline covariates. We included 39 variables encompassing demographic characteristics, clinical measurements, comorbidities and medication history as detailed in Table 1, together with geographical region and year of trial enrolment. Covariate selection was informed by prior observational studies in HF^34^.

Missing data were handled using multivariate imputation by chained equations (MICE), generating five imputed datasets over ten iterations^35^. Imputation models included all observed covariates, the mortality outcome, and the logarithm of time-to-event. Treatment effect estimates were calculated separately within each imputed dataset and pooled using Rubin’s rules^36^.

All effect estimates are reported with two-sided 95% confidence intervals. Because the sequential TTE framework allowed individuals to contribute to multiple monthly trials, confidence intervals were estimated using cluster-robust variance estimators clustered at the patient level to account for intra-individual correlation^37^.

#### Propensity score matching

Propensity score matching is a statistical technique commonly used in observational studies to reduce confounding bias when estimating treatment effects^38^. It involves matching treated and untreated individuals based on their propensity score, defined as the probability of receiving treatment conditional on observed baseline covariates. This approach creates a balanced cohort in terms of baseline covariates that mimics randomisation. However, PSM can lead to a reduction in the overall sample size, potentially diminishing the statistical power of the findings, and depending on matching stringency, may shift the target population and reduce generalisability^39^.

In this study, propensity scores were estimated using logistic regression with the pre-specified covariate set described above. Treated individuals were matched 1:1 to controls using nearest-neighbour matching with a caliper of 0.2 standard deviations of the logit of the propensity score. Hazard ratios were then estimated by fitting a Cox proportional hazards regression of mortality on treatment within the matched cohort.

#### Inverse probability of treatment weighting

Inverse probability of treatment weighting adjusts for confounding by assigning weights to individuals based on the inverse of their probability of receiving the treatment they actually received^40^. This weighting creates a pseudo-population in which measured confounders are more balanced across groups, facilitating less biased estimation of treatment effects. A known limitation of IPTW is its sensitivity to extreme propensity scores (close to 0 or 1), which can result in large weights and thus produce unstable estimates with inflated variance.

We estimated the propensity scores via logistic regression, then calculated the IPTW weights. To enhance weight stability and reduce variance inflation, we applied stabilised IPTW, which multiplies the original IPTW weights by the marginal probability of receiving the observed treatment^41^. Additionally, we implemented Stürmer trimming to exclude individuals with propensity scores below the 1st percentile in the treated group or above the 99th percentile in the control group^16^. Hazard ratios were estimated by fitting a weighted Cox proportional hazards regression of mortality on treatment, with weights set to the stabilised IPTW weights.

#### Targeted maximum likelihood estimation

Targeted maximum likelihood estimation is a doubly robust method that generates treatment effect estimates by fitting both a treatment model and an outcome model, then applying a targeted update to the outcome estimates using the treatment estimates^15^. This targeted step reduces bias in the effect estimate while maintaining control over variance. TMLE is considered doubly robust because it yields consistent estimates if either the treatment model or the outcome model is correctly specified. TMLE’s performance can be compromised when both models are mis-specified.

In our implementation, the treatment model was a logistic regression of treatment on the pre-specified covariate set described above, and the outcome model was a logistic regression of two-year all-cause mortality on treatment and the same covariate set. We applied the same trimming as used for IPTW to maintain consistency across adjustment strategies. To ensure numerical stability during the targeted fluctuation step, propensity scores were bounded at [0.025, 0.975] and initial outcome predictions were bounded at [0.0005, 0.9995]. Risk ratios were estimated as the ratio of the marginal mean updated predicted probabilities of two-year mortality under treatment versus no treatment.

#### Deep learning with TMLE

Transformer-based deep learning models can automatically learn rich, high-dimensional patient representations from raw EHR data^42^, capturing both measured covariates and latent patterns that may reflect unmeasured confounding. The Targeted Bidirectional Electronic Health Records Transformer (T-BEHRT) builds on the Transformer architecture and uses these representations to estimate propensity scores and outcome probabilities^11^. By combining DL-based estimations with TMLE, T-BEHRT eliminates the need for manual covariate selection and provides doubly robust causal estimates using EHR data. It has been applied to assess associations between antihypertensive treatments and cancer, and between paracetamol use and cardiovascular events^43^.

In this study, T-BEHRT estimated treatment and outcome probabilities using health records up to each participant’s baseline date. We applied the same propensity score trimming approach as used for IPTW and utilised cross-validated TMLE to derive risk ratios. Because deep learning models often struggle with highly imbalanced datasets and tend to favour the majority class^17^, we implemented subsampling by randomly selecting 20 controls for each treated patient to improve model training and ensure fair comparisons. Details of model training are provided in **Supplementary Methods: Deep learning**.

### Sensitivity analysis

We performed a range of sensitivity analyses to assess the robustness of our findings. First, we varied the trimming threshold (from the 1st to the 10th percentile) and experimented with different control subsampling ratios (1:5 to 1:20) to evaluate their impact on the effect estimates. Second, there is potential heterogeneity among individuals with and without AF^44^, so we performed stratified analyses by AF status for both drugs. In addition, we visualised the propensity score distributions from both the logistic regression and DL models in probability density plots and compared the covariate profiles before and after matching to aid interpretation.

### Semi-synthetic simulation

To systematically evaluate the robustness of each adjustment method under varying levels of confounding, we performed a semi-synthetic simulation^11^ that generated treatment assignments and outcomes based on the empirical distribution of baseline covariates in the beta-blockers cohort. Five chronic conditions (hypertension, diabetes, ischaemic heart disease, thyroid disorder, and COPD) were specified as confounders. Details of the simulation procedure and the coefficients defining the degree of confounding for each condition are reported in **Supplementary Methods: Simulation**.

To simulate realistic situations in which key clinical variables are unknown or unavailable for adjustment, we intentionally omitted selected confounders in some scenarios. Specifically, we compared all adjustment methods across three settings: (1) two unknown confounders: only covariates from Table 1 were adjusted (omitting COPD and thyroid disorder, which were specified as confounders in the simulation); (2) one unknown confounder: where COPD was included but thyroid disorder was omitted; and (3) no unknown confounding: where all covariates were included. In all scenarios, the deep learning approach used all available pre-baseline EHR data without manual covariate selection. This framework allowed direct evaluation of how each method performs as the extent of confounding increases.

### Reporting summary

Further information on research design is available in the Nature Portfolio Reporting Summary linked to this article.

## Supplementary information

Supplementary information

Reporting Summary

Transparent Peer Review file

## Data Availability

Access to CPRD data is granted only after approval of a study protocol through CPRD’s Research Data Governance Process (https://www.cprd.com/research-applications). These data cannot be shared directly by the authors.

## Code availability

The code for data processing and statistical analysis is available at: https://github.com/Zhengxian-Fan/target-trial-emulation. The code used to train the deep learning model is available at: https://github.com/deepmedicine/Targeted-BEHRT.
