# Supplementary material for: Evaluating bias in target trial emulation for heart failure across statistical and deep learning methods
Source: Nat Commun. 2026 Jul 13;17:8618. doi: 10.1038/s41467-026-74999-6 (PMC13486677; doi:10.1038/s41467-026-74999-6)

Table of Contents

Supplementary Methods ***.. 2***

Supplementary Tables ***.. 4***

Supplementary Table 1. Specification of a target trial protocol for beta-blockers in HFrEF..... 4

Supplementary Table 2. Specification of a target trial protocol for digoxin in HFrEF. 5

Supplementary Table 3. Risk ratios for 2-year all-cause mortality by treatment-to-control sampling ratio using a deep-learning approach under 1st percentile Stürmer trimming......... 6

Supplementary Figures ***.. 7***

Supplementary Figure 1. Propensity score distributions for beta-blockers and digoxin initiation estimated using logistic regression and deep learning. 7

Supplementary Figure 2. Influence of Stürmer trimming thresholds on adjusted effect estimates for initiation of beta-blockers. 8

Supplementary Figure 3. Influence of Stürmer trimming thresholds on adjusted effect estimates for initiation of digoxin.. 9

Supplementary Figure 4. Relative effect estimates of initiating beta-blockers and digoxin on 2-year all-cause mortality in patients with heart failure with reduced ejection fraction (HFrEF), stratified by atrial fibrillation (AF) status. 10

Supplementary Figure 5. Propensity score distributions in the semi-synthetic simulation estimated using logistic regression and deep learning. 11

Supplementary Methods

Deep Learning

The T-BEHRT model estimated treatment effects using both static baseline variables (age, sex, smoking status, and region) and longitudinal electronic health record (EHR) data, including all diagnoses, prescriptions, procedures, and laboratory results recorded before baseline. Model training was performed in two stages. First, the model was trained on the emulated trial cohort using a masked encounter modelling (MEM) objective for 5 epochs. In this step, 15% of encounters within each patient sequence were randomly masked, and the model was trained to predict the original masked encounters from the surrounding sequence context. This self-supervised objective enables the model to learn contextualised representations of patients' longitudinal clinical histories within the study population. Second, the model was optimised for 3 epochs to jointly estimate propensity scores and conditional outcome risks under each exposure status. Training parameters included a batch size of 32, a maximum sequence length of 256, and a learning rate set to 1e-4. A 5-fold cross-validation strategy, consistent with the original T-BEHRT implementation, was used throughout. Predictions of treatment propensity and outcome risk were generated for each held-out test fold and then pooled to estimate the risk ratio.

Simulation

We derived a severity score Z^* based on five chronic conditions: hypertension, diabetes, ischaemic heart disease, thyroid disorder, and chronic obstructive pulmonary disease. Patients with more than two of these conditions were classified as "severely ill" (Z^* = 1); otherwise (Z^* = 0). Treatment A was simulated using a Bernoulli distribution conditioned on Z^*. Severely ill patients (Z^* = 1) had a 90% probability of receiving treatment (A = 1), whereas less severely ill patients (Z^* = 0) had a 10% probability. This induces strong confounding between baseline health status and treatment allocation.

Follow-up was discretized into monthly intervals over 24 months. The hazard for patient i at month t was specified as:

$$h_i(t) = H_0 e^{-\lambda t} \cdot e^{\theta A_i} \cdot e^{\sum_{j=1}^{9} \beta_j Z_{ij}} \cdot e^{\log(\gamma) t Z_i^*}$$

Where:

- $H_0 e^{-\lambda t}$ represents the baseline hazard, with H_0 = 0.0001 and λ = 0.25
- θ = -0.5 quantifies the treatment effect
- each static covariate coefficient β_j was drawn from $Uniform\ (0, 0.5)$ and Z_{ij} includes age (scaled to [0,1]), sex, atrial fibrillation, stroke, and the five conditions used to define Z^*.
- γ = 1.01 imposes a 1% per-month hazard increase for severely ill patients, capturing progressive deterioration.

The β_j coefficients used in the simulation (drawn from Uniform(0,0.5)) were:

- Age: 0.324
- Sex: 0.254
- Hypertension: 0.264
- Diabetes: 0.448
- Ischaemic heart disease: 0.350
- Thyroid disorder: 0.357
- Chronic obstructive pulmonary disease: 0.359
- Atrial fibrillation: 0.111
- Stroke: 0.088

At each month t, an event $E_i(t) \sim Bernoulli\left(h_i(t)\right)$ was drawn. The observed time-to-event T_i was defined as the first t with $E_i(t) = 1$. Patients without an event by month 24 were right censored at 24 months.

We performed two counterfactual simulations setting $A = 1$ and $A = 0$ for all patients. For each scenario, we calculated the mean hazard across individuals and time points, then computed:

$$\mathrm{HR} = \frac{E[h_i(t)|A = 1]}{E[h_i(t)|A = 0]}$$

with 95% confidence intervals obtained via nonparametric bootstrap.

Supplementary Tables

Supplementary Table 1. Specification of a target trial protocol for beta-blockers in HFrEF.

Protocol component	Target trial specification	Target trial emulation
**Eligibility criteria**	Adults aged 40–80 years with HFrEF and NYHA class II–IV, receiving optimal background therapy.	Patients aged 40–80 years with HFrEF receiving usual care.
**Treatment strategies**	Initiation of beta-blockers versus placebo.	Initiation of beta-blockers versus no initiation (continuation of usual care) within the month of enrolment.
**Treatment assignment**	Randomised, double-blind allocation.	Patients were assigned to the strategy they received at baseline; randomisation was emulated through adjustment for baseline confounders.
**Outcomes**	All-cause mortality.	Same as for the target trial.
**Follow-up**	From randomisation to death, loss to follow-up, or study completion, whichever occurred first.	From baseline to death, loss to follow-up (practice deregistration), or completion of the 2-year follow-up period, whichever occurred first.
**Causal contrast**	Intention-to-treat (ITT) effect.	Observational analogue of the ITT effect

HFrEF = heart failure with reduced ejection fraction; NYHA = New York Heart Association

Supplementary Table 2. Specification of a target trial protocol for digoxin in HFrEF.

Protocol component	Target trial specification	Target trial emulation
**Eligibility criteria**	Adults with HFrEF in normal sinus rhythm, receiving optimal background therapy.	Patients aged 35–85 years with HFrEF receiving usual care.
**Treatment strategies**	Initiation of digoxin versus placebo.	Initiation of digoxin versus no initiation (continuation of usual care) within the month of enrolment.
**Treatment assignment**	Randomised, double-blind allocation.	Patients were assigned to the strategy they received at baseline; randomisation was emulated through adjustment for baseline confounders.
**Outcomes**	All-cause mortality.	Same as for the target trial.
**Follow-up**	From randomisation to death, loss to follow-up, or study completion, whichever occurred first.	From baseline to death, loss to follow-up (practice deregistration), or completion of the 2-year follow-up period, whichever occurred first.
**Causal contrast**	Intention-to-treat (ITT) effect.	Observational analogue of the ITT effect.

HFrEF = heart failure with reduced ejection fraction

Supplementary Table 3. Risk ratios for 2-year all-cause mortality by treatment-to-control sampling ratio using a deep-learning approach under 1st percentile Stürmer trimming.

**Sampling Ratio**<br><br>**(Treatment: Control)**	**Beta-blockers**<br><br>**RR (95% CI)**	**Digoxin**<br><br>**RR (95% CI)**
**1:5**	1.07 (0.98–1.16)	1.32 (1.19–1.45)
**1:10**	1.13 (1.05–1.23)	1.49 (1.34–1.66)
**1:15**	1.14 (1.04–1.27)	1.43 (1.30–1.59)
**1:20**	1.16 (1.03–1.28)	1.44 (1.30–1.59)

Abbreviations: RR, risk ratio; CI, confidence interval. Note: Treatment: control ratio indicates the number of control subjects sampled per treatment subject.

Supplementary Figures

Supplementary Figure 1. Propensity score distributions for beta-blockers and digoxin initiation estimated using logistic regression and deep learning.

Density plots show estimated treatment propensity scores for treated and control individuals. The overlap coefficient quantifies similarity between the two distributions, with lower values indicating reduced overlap and greater separation.

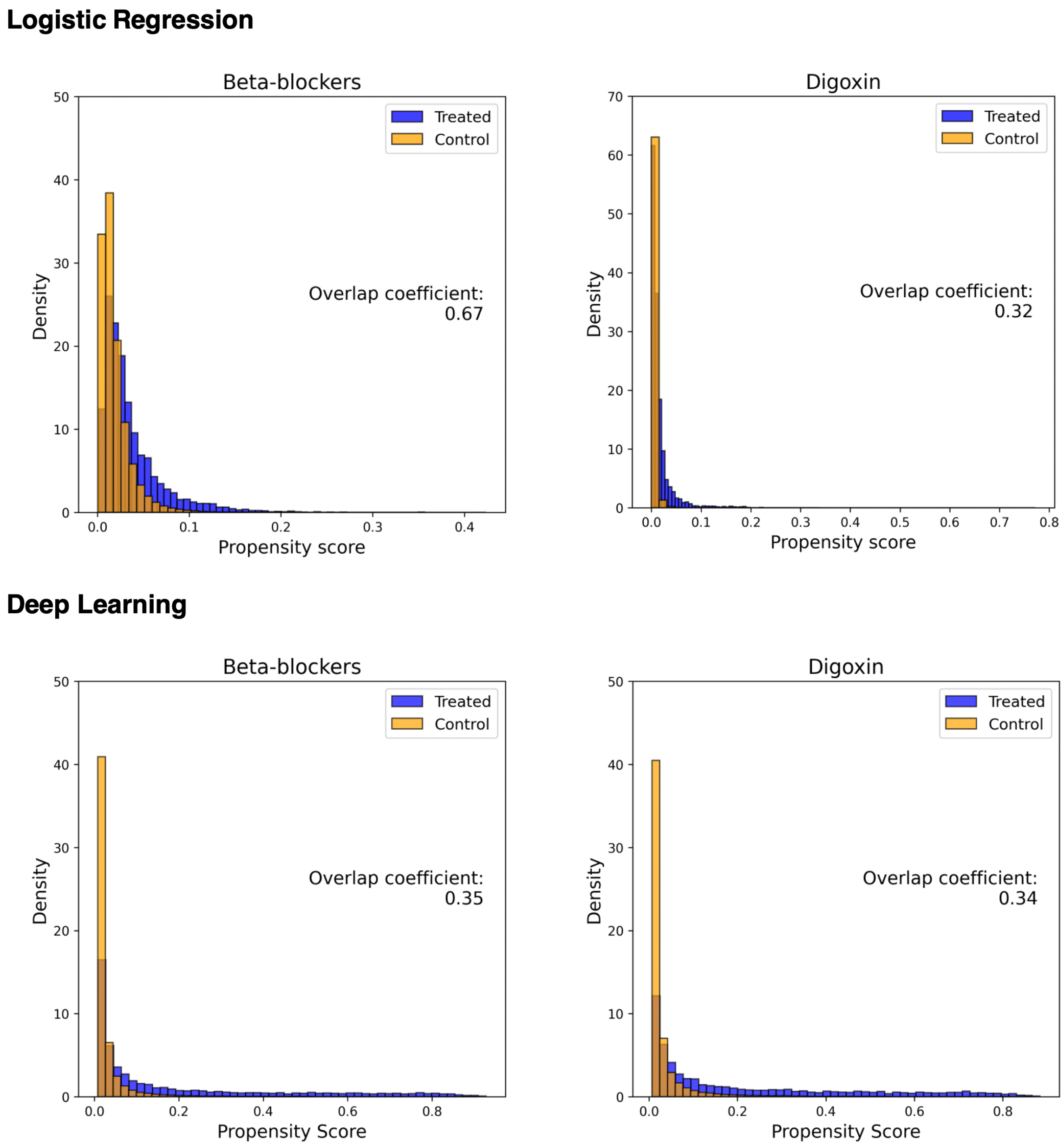

Supplementary Figure 2. Influence of Stürmer trimming thresholds on adjusted effect estimates for initiation of beta-blockers.

Effect estimates from inverse probability of treatment weighting (IPTW), targeted maximum likelihood estimation (TMLE), and deep learning are shown across trimming thresholds from 1% to 10%. A trimming threshold of 1% removes treated individuals below the 1st percentile of the treated propensity score distribution and control individuals above the 99th percentile of the control distribution. Error bars represent 95% confidence intervals (CI).

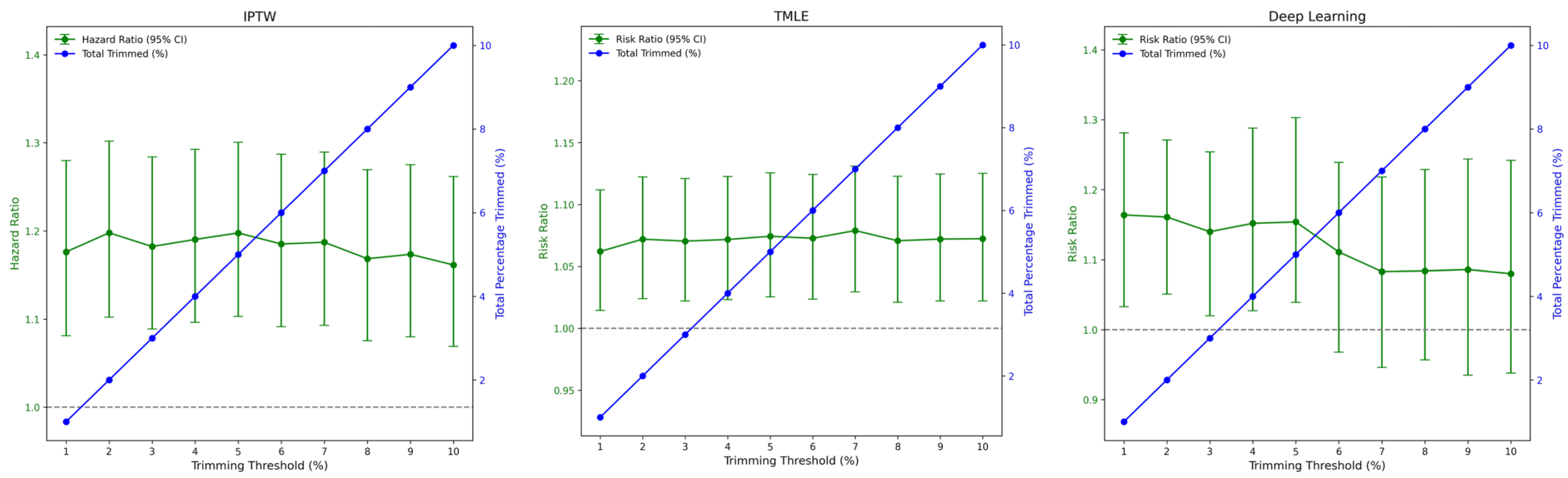

Supplementary Figure 3. Influence of Stürmer trimming thresholds on adjusted effect estimates for initiation of digoxin.

Effect estimates from inverse probability of treatment weighting (IPTW), targeted maximum likelihood estimation (TMLE), and deep learning are shown across trimming thresholds from 1% to 10%. A trimming threshold of 1% removes treated individuals below the 1st percentile of the treated propensity score distribution and control individuals above the 99th percentile of the control distribution. Error bars represent 95% confidence intervals (CI).

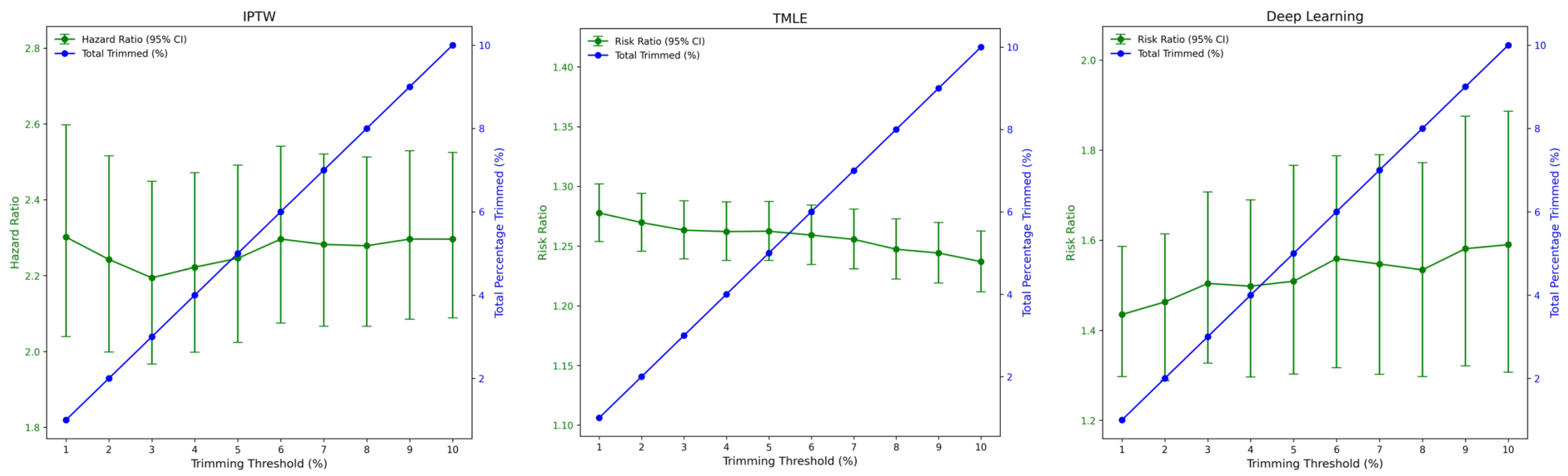

Supplementary Figure 4. Relative effect estimates of initiating beta-blockers and digoxin on 2-year all-cause mortality in patients with heart failure with reduced ejection fraction (HFrEF), stratified by atrial fibrillation (AF) status.

Estimates from crude analyses, propensity score matching (PSM), and inverse probability of treatment weighting (IPTW) are presented as hazard ratios; estimates from targeted maximum likelihood estimation (TMLE) and deep learning (DL) are presented as risk ratios. Squares represent point estimates and error bars represent 95% confidence intervals (CI). n, number of deaths; N, group size.

Method	Total number of participants	Initiators (n/N)	Non-Initiators (n/N)	Effect Estimate (95% CI)
**Beta-blockers (with AF)**				
Crude	155,741	506 / 3,273	22,264 / 152,468	1.09 (0.99–1.20)
PSM	6,546	506 / 3,273	527 / 3,273	0.98 (0.86–1.12)
IPTW	154,183	497 / 3,240	21,949 / 150,943	1.16 (1.03–1.30)
TMLE	154,183	497 / 3,240	21,949 / 150,943	1.02 (0.95–1.09)
DL	62,764	501 / 3,235	8,576 / 59,529	1.13 (0.97–1.27)
**Beta-blockers (without AF)**				
Crude	261,589	488 / 4,803	23,602 / 256,786	1.13 (1.03–1.25)
PSM	9,604	487 / 4,802	492 / 4,802	1.01 (0.87–1.16)
IPTW	258,972	479 / 4,754	23,318 / 254,218	1.15 (1.01–1.30)
TMLE	258,972	479 / 4,754	23,318 / 254,218	1.09 (1.03–1.17)
DL	104,927	486 / 4,750	9,112 / 100,177	1.18 (0.97–1.35)
**Digoxin (with AF)**				
Crude	579,799	750 / 3,805	82,808 / 575,994	1.47 (1.36–1.58)
PSM	7,610	750 / 3,805	669 / 3,805	1.17 (1.05–1.30)
IPTW	574,000	743 / 3,766	81,597 / 570,234	1.43 (1.29–1.58)
TMLE	574,000	743 / 3,766	81,597 / 570,234	1.21 (1.18–1.25)
DL	33,615	744 / 3,762	4,199 / 29,853	1.16 (1.07–1.25)
**Digoxin (without AF)**				
Crude	1,098,404	157 / 572	103,617 / 1,097,832	3.50 (2.97–4.13)
PSM	1,144	157 / 572	88 / 572	2.02 (1.57–2.61)
IPTW	1,087,419	155 / 566	101,528 / 1,086,853	2.46 (1.86–3.25)
TMLE	1,087,419	155 / 566	101,528 / 1,086,853	1.63 (1.59–1.67)
DL	57,236	156 / 564	5,309 / 56,672	1.89 (1.47–2.31)

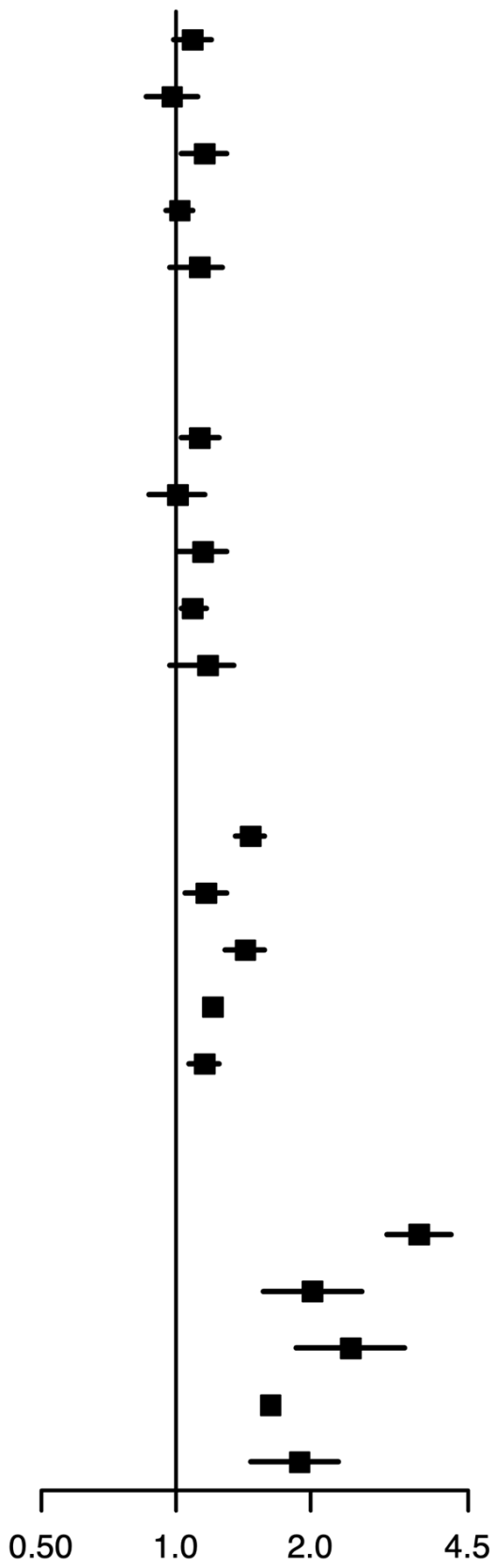

Supplementary Figure 5. Propensity score distributions in the semi-synthetic simulation estimated using logistic regression and deep learning. Density plots show estimated treatment propensity scores for treated and control individuals. The overlap coefficient quantifies similarity between the two distributions, with lower values indicating reduced overlap and greater separation.

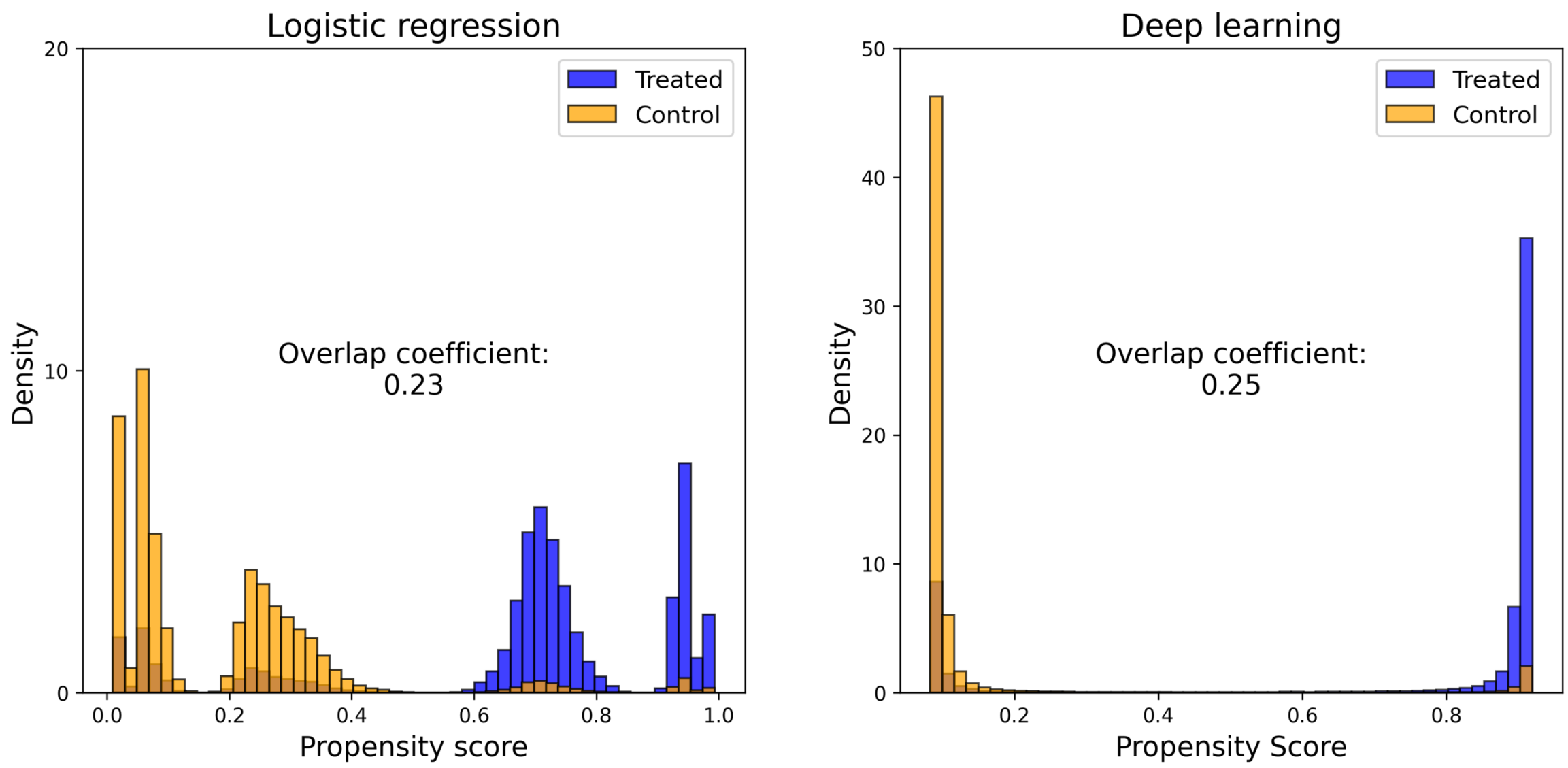

Supplement: Supplementary file 1 [file 41467_2026_74999_MOESM1_ESM.pdf]
