# Supplementary material for: Evaluating bias in target trial emulation for heart failure across statistical and deep learning methods
Source: Nat Commun. 2026 Jul 13;17:8618. doi: 10.1038/s41467-026-74999-6 (PMC13486677; doi:10.1038/s41467-026-74999-6)
Supplement: Supplementary file 2 [file 41467_2026_74999_MOESM2_ESM.pdf]

nature portfolio

Corresponding author(s): Shishir Rao, Kazem Rahimi

Last updated by author(s): May 13, 2026

Reporting Summary

Nature Portfolio wishes to improve the reproducibility of the work that we publish. This form provides structure for consistency and transparency in reporting. For further information on Nature Portfolio policies, see our Editorial Policies and the Editorial Policy Checklist.

Statistics

For all statistical analyses, confirm that the following items are present in the figure legend, table legend, main text, or Methods section.

n/a	Confirmed	
☐	☒	The exact sample size (*n*) for each experimental group/condition, given as a discrete number and unit of measurement
☐	☒	A statement on whether measurements were taken from distinct samples or whether the same sample was measured repeatedly
☐	☒	The statistical test(s) used AND whether they are one- or two-sided<br>*Only common tests should be described solely by name; describe more complex techniques in the Methods section.*
☐	☒	A description of all covariates tested
☐	☒	A description of any assumptions or corrections, such as tests of normality and adjustment for multiple comparisons
☐	☒	A full description of the statistical parameters including central tendency (e.g. means) or other basic estimates (e.g. regression coefficient) AND variation (e.g. standard deviation) or associated estimates of uncertainty (e.g. confidence intervals)
☐	☒	For null hypothesis testing, the test statistic (e.g. *F*, *t*, *r*) with confidence intervals, effect sizes, degrees of freedom and *P* value noted<br>*Give P values as exact values whenever suitable.*
☒	☐	For Bayesian analysis, information on the choice of priors and Markov chain Monte Carlo settings
☒	☐	For hierarchical and complex designs, identification of the appropriate level for tests and full reporting of outcomes
☐	☒	Estimates of effect sizes (e.g. Cohen's *d*, Pearson's *r*), indicating how they were calculated

Our web collection on statistics for biologists contains articles on many of the points above.

Software and code

Policy information about availability of computer code

Data collection	CPRD collects anonymised, longitudinal primary care data from a network of GP practices across the UK.
Data analysis	Code for processing the data and conducting the analyses is available at https://github.com/Zhengxian-Fan/target-trial-emulation. Analyses were performed in Python (≥3.8) using pyspark (≥3.2.0), pandas (≥1.3.0), numpy (≥1.21.0), scipy (≥1.7.0), scikit-learn (≥1.0.0), lifelines (≥0.26.0), and joblib (≥1.1.0), and in R (≥4.0) using arrow (≥6.0.0), tidyverse (≥1.3.0), and mice (≥3.14.0).

For manuscripts utilizing custom algorithms or software that are central to the research but not yet described in published literature, software must be made available to editors and reviewers. We strongly encourage code deposition in a community repository (e.g. GitHub). See the Nature Portfolio guidelines for submitting code & software for further information.

Data

Policy information about availability of data

All manuscripts must include a data availability statement. This statement should provide the following information, where applicable:

- Accession codes, unique identifiers, or web links for publicly available datasets
- A description of any restrictions on data availability
- For clinical datasets or third party data, please ensure that the statement adheres to our policy

Access to CPRD data is granted only after approval of a study protocol through CPRD's Research Data Governance Process (https://www.cprd.com/research-applications).

Research involving human participants, their data, or biological material

Policy information about studies with human participants or human data. See also policy information about sex, gender (identity/presentation), and sexual orientation and race, ethnicity and racism.

Reporting on sex and gender	Sex was identified based on the sex recorded in primary care and secondary care records as captured in CPRD Aurum. Sex was considered in the study design and included as a baseline covariate in propensity score matching, inverse probability of treatment weighting, targeted maximum likelihood estimation, and the deep learning model. The study included both female and male participants; sex composition by treatment group is reported in the baseline characteristics table. Pre-specified sex-disaggregated effect estimates were not conducted because the primary objective concerned methodological performance rather than effect heterogeneity, and disaggregation would have reduced statistical power within the initiator subgroups.
Reporting on race, ethnicity, or other socially relevant groupings	Socioeconomic status (measured by the Index of Multiple Deprivation quintiles) was used as a socially relevant grouping variable and was included as a covariate in the analyses to control for confounding. Ethnicity (categorised as White, South Asian, Black, Other, and Unknown) was reported in the baseline characteristics table and included as a covariate. These variables were derived from routine electronic health records.
Population characteristics	The study population comprised individuals with a recorded diagnosis of heart failure with reduced ejection fraction (HFrEF). Covariates extracted from electronic health records included demographic characteristics (age, sex, smoking status, and socioeconomic status), comorbidities (e.g., hypertension, diabetes, ischaemic heart disease, atrial fibrillation, asthma, and chronic obstructive pulmonary disease), clinical measurements (e.g., body mass index and systolic blood pressure), and prior medication use (e.g., ACE inhibitors, diuretics, anticoagulants, and statins). These variables were used to estimate propensity scores and adjust for confounding.
Recruitment	This study did not actively recruit participants but utilised retrospective observational data sourced from the CPRD Aurum, a primary care database covering approximately 20% of the UK population. The data includes comprehensive demographics, diagnoses, prescriptions, and is linked to Hospital Episode Statistics and Office for National Statistics mortality records.
Ethics oversight	The Clinical Practice Research Datalink (CPRD) Group holds ethical approval from the National Research Ethics Service Committee for all observational studies conducted using CPRD data. This specific study received scientific approval from the CPRD Independent Scientific Advisory Committee (protocol number: 20_095).

Note that full information on the approval of the study protocol must also be provided in the manuscript.

Field-specific reporting

Please select the one below that is the best fit for your research. If you are not sure, read the appropriate sections before making your selection.

☒ Life sciences ☐ Behavioural & social sciences ☐ Ecological, evolutionary & environmental sciences

For a reference copy of the document with all sections, see nature.com/documents/nr-reporting-summary-flat.pdf

Life sciences study design

All studies must disclose on these points even when the disclosure is negative.

Sample size	Sample size was determined by the number of subjects meeting the inclusion criteria in the CPRD Aurum database between January 1, 2010 and January 1, 2015. CPRD Aurum provides a broadly representative sample of the UK population, covering approximately 20% of the population through a network of general practices, and is therefore considered adequate for the analyses presented.
Data exclusions	Patients were excluded if they were aged outside the prespecified age range, had less than one year of registration with their general practice, or had a prescription for the target drug in the year prior to enrolment.
Replication	This study emulated a series of monthly hypothetical trials over a 60-month period rather than relying on a single enrolment point in time. Robustness of findings was assessed through prespecified sensitivity analyses: varying the Stürmer trimming threshold from the 1st to 10th percentile, varying the deep learning subsampling ratio from 1:5 to 1:20. The main finding that no adjustment method reproduced the randomised trial evidence was robust across all analytical variations performed.
Randomization	This was an observational study, so participants were not randomly assigned to treatment groups; instead, assignment was based on observed treatment initiation in routine care. To address confounding by indication, the study used four adjustment approaches.
Blinding	Blinding was not applicable as this was an analysis of electronic health records.

Reporting for specific materials, systems and methods

We require information from authors about some types of materials, experimental systems and methods used in many studies. Here, indicate whether each material, system or method listed is relevant to your study. If you are not sure if a list item applies to your research, read the appropriate section before selecting a response.

Materials & experimental systems

n/a	Involved in the study
☒	☐ Antibodies
☒	☐ Eukaryotic cell lines
☒	☐ Palaeontology and archaeology
☒	☐ Animals and other organisms
☒	☐ Clinical data
☒	☐ Dual use research of concern
☒	☐ Plants

Methods

n/a	Involved in the study
☒	☐ ChIP-seq
☒	☐ Flow cytometry
☒	☐ MRI-based neuroimaging

Plants

Seed stocks

Report on the source of all seed stocks or other plant material used. If applicable, state the seed stock centre and catalogue number. If plant specimens were collected from the field, describe the collection location, date and sampling procedures.

Novel plant genotypes

Describe the methods by which all novel plant genotypes were produced. This includes those generated by transgenic approaches, gene editing, chemical/radiation-based mutagenesis and hybridization. For transgenic lines, describe the transformation method, the number of independent lines analyzed and the generation upon which experiments were performed. For gene-edited lines, describe the editor used, the endogenous sequence targeted for editing, the targeting guide RNA sequence (if applicable) and how the editor was applied.

Authentication

Describe any authentication procedures for each seed stock used or novel genotype generated. Describe any experiments used to assess the effect of a mutation and, where applicable, how potential secondary effects (e.g. second site T-DNA insertions, mosiacism, off-target gene editing) were examined.
