# Supplementary material for: Evaluating bias in target trial emulation for heart failure across statistical and deep learning methods
Source: Nat Commun. 2026 Jul 13;17:8618. doi: 10.1038/s41467-026-74999-6 (PMC13486677; doi:10.1038/s41467-026-74999-6)
Supplement: Supplementary file 3 [file 41467_2026_74999_MOESM3_ESM.pdf]

nature portfolio

Peer Review File

Evaluating bias in target trial emulation for heart failure across statistical and deep learning methods

Corresponding Author: Dr Shishir Rao

This file contains all reviewer reports in order by version, followed by all author rebuttals in order by version.

Version 0:

Reviewer comments:

Reviewer #1

(Remarks to the Author)
This is an important study highlighting the persisting biases in estimating treatment effects using real world evidence data. Despite several adjustment models, the observed effect was not able to reproduce randomized data.
Some points need to be considered:
1) all-cause mortality is quite an ambitious outcome. What about adding CV hospitalization? HF hospitalization?
2) any prespecified protocol for emulating this trial?
3) new randomized trial was published with digoxin and showing some benefits in HF patients, please update the context
4) some subgroups can be interesting to explore to detect any treatment modification including interactions in the model
5) why not adding other recommended drugs (SGLT2, aldaosterome, ARNI) ?

Reviewer #2

(Remarks to the Author)
I appreciate the opportunity to review this manuscript, which tackles an important and timely question. However, I have several concerns, including the framing of discordance with RCT benchmarks as a limitation of the target trial emulation (TTE) framework rather than as a likely consequence of data limitations, such as unmeasured clinical severity, and the interpretability of the method comparisons given that the approaches appear to target different estimands.

Major Comments
Comment 1: The authors frame their study around whether advanced adjustment methods can mitigate residual bias such as confounding by indication. In its current form, this framing is problematic and the novelty is unclear. Under the standard identifying assumption used by these approaches, conditional exchangeability requires that the relevant confounders are measured or adequately proxied. As widely acknowledged in the TTE literature, including by Hernán and colleagues, TTE prevents design-related biases but cannot correct for unmeasured confounding arising from data limitations. Therefore, if crucial clinical severity information (e.g., NYHA class) is absent and not recoverable through strong proxies, persistence of bias is not a surprising result and should not be interpreted as evidence against TTE per se.

Comment 2: The authors state in the Introduction that “propensity score matching (PSM) has been the main method for confounding adjustment.” This is overstated. PSM is a common approach, but weighting-based methods are also widely used in modern pharmacoepidemiology. The text should be revised to describe PSM as “a common method” and to acknowledge the broader set of routinely applied approaches.

Comment 3: The manuscript exhibits a fundamental flaw in the hierarchy of study design: it allows the statistical method to dictate the estimand, rather than defining the estimand to guide the method selection. The causal estimand (e.g., average treatment effect [ATE] vs. average treatment effect on the treated [ATT]) must be defined a priori based on the research question. The statistical method should then be chosen to target that specific estimand. The authors compare PSM, which typically estimates the ATT (or a specific matched subset), against inverse probability treatment weighting (IPTW) and TMLE, which typically estimate the ATE. Since these methods target different populations (a matched subset vs. the entire eligible population), it is expected that their estimates will differ. Treating these differences solely as “methodological performance” without harmonizing the target populations (estimands) is invalid. The authors must explicitly define their target estimand (ATE or ATT) first, and then ensure all applied methods (including deep learning) are configured to estimate that

same quantity to allow for a valid comparison.

Comment 4: There is an unexplained mismatch between the hypothetical target trial and the emulated trial (Supplementary Table 1). The target trial specifies eligibility as “Age 40–80,” yet the emulation includes “Age 35–85.” A discordance in results between the target trial specification and emulation could simply be due to this difference in population, rather than TTE framework failure. The authors must explain this deviation or re-run the analysis with the correct age restrictions to ensure a valid emulation.

Comment 5: The authors constructed “monthly hypothetical trials.” It is unclear how they handled the correlation of data for patients who were eligible in multiple consecutive months. If the same patient appears in multiple "trials" without appropriate variance adjustment, such as robust sandwich estimators or bootstrap methods, the confidence intervals may be artificially narrow.

Reviewer #3

(Remarks to the Author)
I have read with great interest the manuscript entitled " Evaluating bias in target trial emulation of heart failure therapies: a comparison of classical and deep learning adjustment methods" submitted to the Nature Communications.
This original study selected patients with heart failure with reduced ejection fraction and emulated target trials initiated monthly from January 1, 24 2010, to January 1, 2015, to assess the effects of β blockers (positive control) and digoxin 25 (negative control) versus usual care on two-year all-cause mortality. They applied four adjustment strategies: propensity score matching (PSM), inverse probability of treatment weighting, targeted maximum likelihood estimation, and a transformer based deep learning approach, benchmarking estimates against RCT findings.
The authors should be congratulated for performing such a novel and comparative analysis. While the manuscript is well-structured and insightful, it has several methodological, reporting, and interpretive limitations, that should be aknowledged.
1. Please the authors to add a reference in the first paragraph of Introduction
2. While the authors recognize the limitations of TTE, they have not emphasized on the positive aspects of this approach. It should be clarified in the second paragraph of Introduction.
3. Line 93: How is usual care defined?
4. Lines 95-97: Digoxin has not shown benefit in patients with HFrEF, but neither harm. Could it be included as negative control?
a. Recently, a RCT showed that digitoxin led to a lower combined risk of death from any cause or hospital admission for worsening heart failure than placebo among patients with heart failure and reduced ejection fraction who received guideline-directed medical therapy. (10.1056/NEJMoa2415471). How might this affect the selection of digoxin as negative control.
5. Have the authors pre-registered the protocol of their study? If yes, it should be acknowledged.
6. Line 125: Why the patients should be registered with a GP for at least two years before enrolment?
7. Why did the authors select a single outcome of their analysis?
8. Lines 157, 171, 184. The abbreviations should be avoided in the start of each sentence.
9. Table 1.
a. Dapaglifozin and Entresto should have been included in the Medications, considering that both are first-line treatment for HFrEF.
b. Table 1. There are no benign malignancies.
c. Except systolic and diastolic BP, I would also HR as it plays important role in the prognosis of such patients.
10. Lines 252-253: Does the adherence in beta-blockers and digoxin could affect the results?
11. Table 2: Valve disorders were not selected in the prespecified variables, as presented in Table 1.
12. As presented in Figure 2, discrepancies in beta-blockers are smaller compared to Digoxin. How is this explained?
13. The authors should add in the Discussion how their study in the current research practice and which method to be selected.

Reviewer #4

(Remarks to the Author)
I co-reviewed this manuscript with one of the reviewers who provided the listed reports. This is part of the Nature Communications initiative to facilitate training in peer review and to provide appropriate recognition for Early Career Researchers who co-review manuscripts.

Version 1:

Reviewer comments:

Reviewer #1

(Remarks to the Author)
The answers were well-drafted although finally there is only a minor improvement. New drugs were not included in the analysis. The methodology is good but the clinical significance remains limited for practice.

Reviewer #2

(Remarks to the Author)
The authors have addressed the previous comments appropriately, and I have no further comments.

Reviewer #3

(Remarks to the Author)
I thank the authors for their thorough and detailed response to the reviewers' comments. The revision is substantive and demonstrates genuine engagement with the concerns raised. The manuscript has improved considerably, and I am broadly satisfied that the major concerns have been addressed. However, several residual issues require attention before further evaluation. More specifically;
1. Reviewer 1 asked the authors to explore subgroups to detect treatment effect modification, "including interactions in the model." The authors responded with AF-stratified analyses only. While the AF stratification is clinically relevant and well-presented, the response does not acknowledge the narrowness of this choice. In a study of this scale, with >400,000 participants and 39 baseline covariates, the absence of any exploration of other clinically relevant effect modifiers (e.g., age, sex, renal function, baseline heart rate, diabetes status) is notable. I recognise that the primary emulation failed to replicate the main RCT effect, which limits the interpretability of subgroup findings. However, this reasoning should be stated explicitly in the reviewer's response. A brief statement in the Discussion acknowledging that broader subgroup analyses were not performed and explaining why would be appropriate.
2. The response provides a reasonable definition of "usual care" as routine primary care management. However, this definition does not appear to have been incorporated into the manuscript itself. The Methods section and Results section refer to "usual care" without explicit definition. Since readers of the published manuscript will not have access to the rebuttal, this definition should be added to the Methods.
3. The authors commendably re-extracted the beta-blocker cohort with 40–80 years to match the benchmark RCT. However, the Methods (line 395–396) still state that the digoxin cohort uses an age range of 35–85 years. The DIG trial enrolled patients aged 21 and over. The rebuttal does not explicitly clarify whether Reviewer 2's concern applied only to the beta-blocker cohort or to both cohorts. For transparency, the authors should add a brief justification for the 35–85 age restriction in the digoxin emulation, explaining how it relates to the DIG trial's eligibility criteria and whether this narrower range might itself contribute to discordance with the original trial estimates.
4. The Discussion briefly mentions that active-comparator designs "may help alleviate some of these imbalances" (lines 319–321). Given the central finding that confounding by indication drives the discordance, particularly for digoxin, I would encourage the authors to expand this point. Specifically, how might the study conclusions differ if the authors had employed an active-comparator new-user design (e.g., beta-blocker vs. ACE inhibitor initiation, or digoxin vs. another rate-control agent)?

Reviewer #4

(Remarks to the Author)
I co-reviewed this manuscript with one of the reviewers who provided the listed reports. This is part of the Nature Communications initiative to facilitate training in peer review and to provide appropriate recognition for Early Career Researchers who co-review manuscripts.

Version 2:

Reviewer comments:

Reviewer #3

(Remarks to the Author)
The authors have incorporated reviewers #3 comments.
Nothing else to comment on.

Open Access This Peer Review File is licensed under a Creative Commons Attribution 4.0 International License, which permits use, sharing, adaptation, distribution and reproduction in any medium or format, as long as you give appropriate credit to the original author(s) and the source, provide a link to the Creative Commons license, and indicate if changes were made.
In cases where reviewers are anonymous, credit should be given to 'Anonymous Referee' and the source.
The images or other third party material in this Peer Review File are included in the article's Creative Commons license, unless indicated otherwise in a credit line to the material. If material is not included in the article's Creative Commons license and your intended use is not permitted by statutory regulation or exceeds the permitted use, you will need to obtain permission directly from the copyright holder.
To view a copy of this license, visit https://creativecommons.org/licenses/by/4.0/

Reviewers’ Comments

Reviewer #1 (Remarks to the Author):

This is an important study highlighting the persisting biases in estimating treatment effects using real world evidence data. Despite several adjustment models, the observed effect was not able to reproduce randomized data.
Some points need to be considered:

1) all-cause mortality is quite an ambitious outcome. What about adding CV hospitalization? HF hospitalization?

Thank you for raising this point. Unlike more recent heart failure trials that use cardiovascular mortality as the primary endpoint, the two trials we are emulating used all-cause mortality as their primary endpoint. Therefore, we chose to focus on replicating the primary endpoint. As we were unable to replicate the primary RCT outcome, we believe there is limited value in reporting additional secondary endpoints, as these results could be misleading in the absence of successful replication of the primary outcome. We also attempted to replicate the heart failure hospitalisation benefit for beta-blockers but were similarly unable to reproduce the trial findings.

2) any prespecified protocol for emulating this trial?

Yes. The target trial specifications and their corresponding emulation details are provided in **Supplementary Tables 1 and 2**.

Protocol component	Target trial specification	Target trial emulation
**Eligibility criteria**	Adults aged 40–80 years with HFrEF and NYHA class II–IV, receiving optimal background therapy.	Patients aged 40–80 years with HFrEF receiving usual care.
**Treatment strategies**	Initiation of beta-blockers versus placebo.	Initiation of beta-blockers versus no initiation (continuation of usual care) within the month of enrolment.
**Treatment assignment**	Randomised, double-blind allocation.	Patients were assigned to the strategy they received at baseline; randomisation was emulated through adjustment for baseline confounders.
**Outcomes**	All-cause mortality.	Same as for the target trial.
**Follow-up**	From randomisation to death, loss to follow-up, or study completion, whichever occurred first.	From baseline to death, loss to follow-up (practice deregistration), or completion of the 2-year follow-up period, whichever occurred first.

Protocol component	Target trial specification	Target trial emulation
**Causal contrast**	Intention-to-treat (ITT) effect.	Observational analogue of the ITT effect

3) new randomized trial was published with digoxin and showing some benefits in HF patients, please update the context

Thank you for raising this important point. We agree that the recent DIGIT-HF trial evaluating digitoxin is highly relevant and should be discussed. We have now incorporated the following paragraph into the Discussion section:

"Digoxin was selected as a negative control on the basis of prior randomised trial evidence indicating no effect on all-cause mortality. More recently, the DIGIT-HF trial evaluated digitoxin, a closely related cardiac glycoside, compared with placebo. The trial reported a reduction in the composite endpoint of death or first hospitalisation for worsening HF, but no clear reduction in all-cause mortality (HR 0.86, 95% CI 0.69–1.07). Taken together, these findings suggest that cardiac glycosides may reduce HF morbidity without clear evidence of harm with respect to mortality. In contrast, our observational analyses consistently yielded elevated mortality estimates for digoxin. The divergence between contemporary trial findings and routine-care estimates reinforces the importance of randomised evidence in this setting and highlights that, even with advanced adjustment methods and comprehensive longitudinal data, deriving reliable treatment effect estimates remains challenging in complex HF populations."

4) some subgroups can be interesting to explore to detect any treatment modification including interactions in the model

We conducted prespecified subgroup analyses stratified by baseline atrial fibrillation (AF) status, given its clinical relevance in heart failure populations. These results are presented in **Supplementary Figure 4** (also attached below).

Method	Total number of participants	Initiators (n/N)	Non-Initiators (n/N)	Effect Estimate (95% CI)
**Beta-blockers (with AF)**				
Crude	155,741	506 / 3,273	22,264 / 152,468	1.09 (0.99–1.20)
PSM	6,546	506 / 3,273	527 / 3,273	0.98 (0.86–1.12)
IPTW	154,183	497 / 3,240	21,949 / 150,943	1.16 (1.03–1.30)
TMLE	154,183	497 / 3,240	21,949 / 150,943	1.02 (0.95–1.09)
DL	62,764	501 / 3,235	8,576 / 59,529	1.13 (0.97–1.27)
**Beta-blockers (without AF)**				
Crude	261,589	488 / 4,803	23,602 / 256,786	1.13 (1.03–1.25)
PSM	9,604	487 / 4,802	492 / 4,802	1.01 (0.87–1.16)
IPTW	258,972	479 / 4,754	23,318 / 254,218	1.15 (1.01–1.30)
TMLE	258,972	479 / 4,754	23,318 / 254,218	1.09 (1.03–1.17)
DL	104,927	486 / 4,750	9,112 / 100,177	1.18 (0.97–1.35)
**Digoxin (with AF)**				
Crude	579,799	750 / 3,805	82,808 / 575,994	1.47 (1.36–1.58)
PSM	7,610	750 / 3,805	669 / 3,805	1.17 (1.05–1.30)
IPTW	574,000	743 / 3,766	81,597 / 570,234	1.43 (1.29–1.58)
TMLE	574,000	743 / 3,766	81,597 / 570,234	1.21 (1.18–1.25)
DL	33,615	744 / 3,762	4,199 / 29,853	1.16 (1.07–1.25)
**Digoxin (without AF)**				
Crude	1,098,404	157 / 572	103,617 / 1,097,832	3.50 (2.97–4.13)
PSM	1,144	157 / 572	88 / 572	2.02 (1.57–2.61)
IPTW	1,087,419	155 / 566	101,528 / 1,086,853	2.46 (1.86–3.25)
TMLE	1,087,419	155 / 566	101,528 / 1,086,853	1.63 (1.59–1.67)
DL	57,236	156 / 564	5,309 / 56,672	1.89 (1.47–2.31)

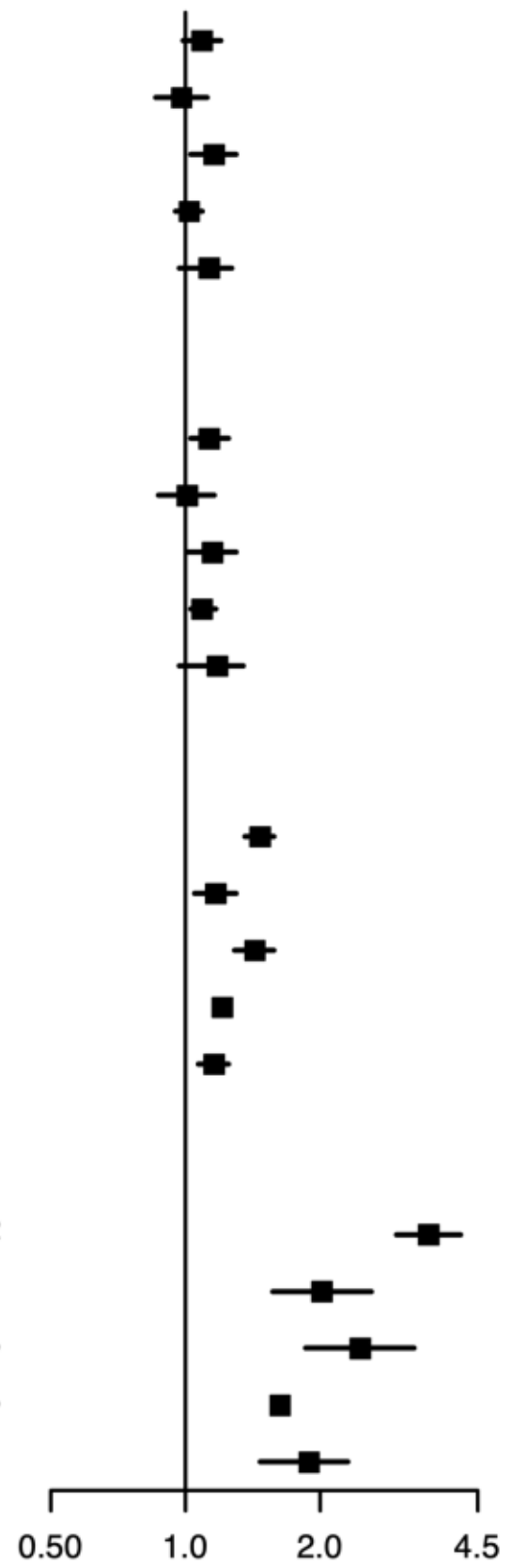

However, because the emulation did not replicate the primary RCT outcomes, we believe that subgroup findings should be interpreted cautiously. For this reason, we only briefly mention these findings in the Results section as follows:

"In analyses stratified by baseline AF status, beta-blocker estimates were broadly similar among individuals with and without AF. For digoxin, all adjustment methods showed elevated mortality risk irrespective of AF status; however, the magnitude of association was substantially greater among individuals without AF than among those with AF."

5) why not adding other recommended drugs (SGLT2, aldosterone, ARNI)?

Thank you for this suggestion. We incorporated aldosterone antagonists as covariates in our adjustment models. SGLT2i and ARNi were not included because during our study period (2010–2015) they were not recommended therapies for heart failure, and very few patients in our cohort were receiving them. With respect to emulating trials of these newer agents, beta-blockers already served as a positive control in our analysis. As we were unable to reproduce the established survival benefit of beta-blockers, extending the emulation to additional positive-control therapies would be unlikely to materially alter the central methodological conclusion.

Reviewer #2 (Remarks to the Author):

I appreciate the opportunity to review this manuscript, which tackles an important and timely question. However, I have several concerns, including the framing of discordance with RCT benchmarks as a limitation of the target trial emulation (TTE) framework rather than as a likely consequence of data limitations, such as unmeasured clinical severity, and the interpretability of the method comparisons given that the approaches appear to target different estimands.

Major Comments

Comment 1: The authors frame their study around whether advanced adjustment methods can mitigate residual bias such as confounding by indication. In its current form, this framing is problematic and the novelty is unclear. Under the standard identifying assumption used by these approaches, conditional exchangeability requires that the relevant confounders are measured or adequately proxied. As widely acknowledged in the TTE literature, including by Hernán and colleagues, TTE prevents design-related biases but cannot correct for unmeasured confounding arising from data limitations. Therefore, if crucial clinical severity information (e.g., NYHA class) is absent and not recoverable through strong proxies, persistence of bias is not a surprising result and should not be interpreted as evidence against TTE per se.

We thank the reviewer for this thoughtful and important comment. We agree that, from a methodological perspective, the TTE framework is designed to address structural and design-related biases such as immortal time bias, rather than to eliminate confounding.

However, there is an important gap between this theoretical understanding and how the framework is often interpreted and applied in practice. Many observational studies using TTE proceed on the implicit assumption that adjusting for a sufficiently rich set of available covariates will be adequate to achieve trial-equivalent estimates. This expectation risks underestimating the magnitude and persistence of confounding by indication. As we noted in our Introduction, several well-designed TTE studies produced findings that were later contradicted by subsequent RCTs. For example, the SENIOR-NSTEMI emulation (Kaura et al., *The Lancet*, 2020) reported a 32% survival benefit that was not confirmed by the SENIOR-RITA trial (Kunadian et al., *NEJM*, 2024). Similarly, a TTE evaluating semaglutide (Wang et al., *Alzheimer's & Dementia*, 2024) suggested a 40–70% reduction in Alzheimer's disease risk, whereas the EVOKE Phase 3 trials (2025) did not demonstrate benefit for disease progression.

The possibility that residual confounding remains despite extensive covariate adjustment is often less visible than design-related bias, and some researchers and regulators may therefore expect that adding more covariates or using more advanced models will necessarily remove it. Our study directly examines that assumption. In addition to commonly used PSM and IPTW, we applied doubly robust TMLE and a deep learning approach combined with full longitudinal EHR data for adjustment. Despite this extensive modelling strategy, the estimated treatment effects remained materially different from those observed in RCTs.

Regarding the reviewer's concern about missing crucial severity information such as NYHA class, we would like to clarify that NYHA is not routinely used to guide heart failure management in UK primary care. Consequently, it is infrequently documented in primary care records, which accounts for the substantial missingness in CPRD and informed our decision not to include it as a covariate. In our recent work using deep learning applied to longitudinal EHR data for heart failure mortality prediction, we demonstrated that the deep learning model achieved a concordance index of 0.845 (95% CI 0.841–0.849), compared with 0.728 (0.723–0.733) for the MAGGIC-HF score, which explicitly incorporates NYHA class and other established severity markers [1]. These findings indicate that longitudinal EHR representations capture substantial prognostic information beyond what is reflected by recorded NYHA status. In other words, heart failure severity appears to be encoded within the latent structure of the EHR to a degree that exceeds the predictive contribution of NYHA alone.

However, despite this ability to capture severity-related prognostic information, the advanced models used in the present study still failed to reproduce RCT estimates. This suggests that the discordance is unlikely to be explained simply by poor data quality or omission of NYHA as a single variable. Rather, it indicates that confounding by indication remains a major source of bias in observational analyses, even when high-dimensional longitudinal data and advanced adjustment methods are employed. These findings highlight the need for caution in placing strong confidence in causal estimates derived under the TTE framework.

[1] Rao, S., Ahmed, N., Salimi-Khorshidi, G., et al. A transformer-based survival model for prediction of all-cause mortality in patients with heart failure: a multi-cohort study. *npj Digital Medicine*. 9, 118 (2026).

Comment 2: The authors state in the Introduction that "propensity score matching (PSM) has been the main method for confounding adjustment." This is overstated. PSM is a common approach, but weighting-based methods are also widely used in modern pharmacoepidemiology. The text should be revised to describe PSM as "a common method" and to acknowledge the broader set of routinely applied approaches.

We agree and have revised the sentence accordingly:

"Common approaches to adjust for confounding include propensity score matching (PSM) and inverse probability of treatment weighting (IPTW)."

Comment 3: The manuscript exhibits a fundamental flaw in the hierarchy of study design: it allows the statistical method to dictate the estimand, rather than defining the estimand to guide the method selection. The causal estimand (e.g., average treatment effect [ATE] vs. average treatment effect on the treated [ATT]) must be defined a priori based on the research question. The statistical method should then be chosen to target that specific estimand. The authors compare PSM, which typically estimates the ATT (or a specific matched subset), against inverse probability treatment weighting (IPTW) and TMLE, which typically estimate the ATE. Since these methods target different

populations (a matched subset vs. the entire eligible population), it is expected that their estimates will differ. Treating these differences solely as "methodological performance" without harmonizing the target populations (estimands) is invalid. The authors must explicitly define their target estimand (ATE or ATT) first, and then ensure all applied methods (including deep learning) are configured to estimate that same quantity to allow for a valid comparison.

We apologise for any confusion caused by our original presentation. We agree that PSM typically estimates the ATT, whereas IPTW, TMLE, and the deep learning approach estimate the ATE. However, our intention was not to directly compare the methods underlying each effect estimate, but rather to compare the findings produced by each method against those from a gold-standard RCT.

It is also common in observational research to report both PSM and IPTW on the same cohort to assess the robustness of findings, even though these methods target different populations and estimands. [1, 2] In such settings, consistency across approaches is interpreted as strengthening causal credibility, despite minor differences in target populations.

We acknowledge that our previous title and parts of the Results section may have unintentionally given the impression that we were comparing methodological performance based on the effect estimates. We have now revised the title and clarified the Results section to eliminate potential confusion.

To further clarify this point, we have added the following statement to the Discussion as a limitation:

"Finally, the estimands differ across methods. PSM restricts the analysis to treated patients and their matched control patients, IPTW and TMLE reweight individuals (with trimming), and the deep learning approach uses subsampling. Consequently, the effect estimates from these approaches are not directly comparable, as each method effectively targets a slightly different population. Differences in effect estimates across methods should therefore not be interpreted as differences in methodological performance, but may in part reflect variation in the underlying target population and potential effect heterogeneity."

References

[1] Zlotta AR, Ballas LK, Niemierko A, et al. Radical cystectomy versus trimodality therapy for muscle-invasive bladder cancer: a multi-institutional propensity score matched and weighted analysis. *Lancet Oncol* 2023;24:669–681. doi:10.1016/S1470-2045(23)00170-5

[2] Hong B, Lee H, Jung K, et al. Sodium-glucose cotransporter-2 inhibitors and risk of autoimmune rheumatic diseases: population based cohort study. *BMJ* 2025;391:e085196. doi:10.1136/bmj-2025-085196

Comment 4: There is an unexplained mismatch between the hypothetical target trial and

the emulated trial (Supplementary Table 1). The target trial specifies eligibility as "Age 40–80," yet the emulation includes "Age 35–85." A discordance in results between the target trial specification and emulation could simply be due to this difference in population, rather than TTE framework failure. The authors must explain this deviation or re-run the analysis with the correct age restrictions to ensure a valid emulation.

Thank you for highlighting this important point. We have now re-extracted the cohort using the age range of 40–80 years to align precisely with the target trial eligibility criteria, re-ran the analyses accordingly, and updated the manuscript. We were still unable to reproduce the trial's reported benefits under the corrected age restriction.

Method	Total number of participants	Initiators (n/N)	Non-Initiators (n/N)	Effect Estimate (95% CI)
**Beta-blockers**				
RCT	3,039	134 / 1,723	159 / 1,316	0.69 (0.54–0.88)
Crude	417,330	994 / 8,076	45,866 / 409,254	1.13 (1.06 - 1.21)
PSM	16,152	994 / 8,076	989 / 8,076	1.03 (0.93 - 1.13)
IPTW	413,158	976 / 7,995	45,287 / 405,163	1.18 (1.08 - 1.28)
TMLE	413,158	976 / 7,995	45,287 / 405,163	1.06 (1.02 - 1.11)
DL	167,691	987 / 7,985	17,687 / 159,706	1.16 (1.03 - 1.28)
**Digoxin**				
RCT	6,800	1,181 / 3,397	1,194 / 3,403	0.99 (0.91–1.07)
Crude	1,678,203	907 / 4,377	186,425 / 1,673,826	2.05 (1.92 - 2.20)
PSM	8,754	907 / 4,377	747 / 4,377	1.29 (1.17 - 1.43)
IPTW	1,661,420	899 / 4,333	183,018 / 1,657,087	2.30 (2.04 - 2.60)
TMLE	1,661,420	899 / 4,333	183,018 / 1,657,087	1.28 (1.25 - 1.30)
DL	90,852	900 / 4,326	9,531 / 86,526	1.44 (1.30 - 1.59)

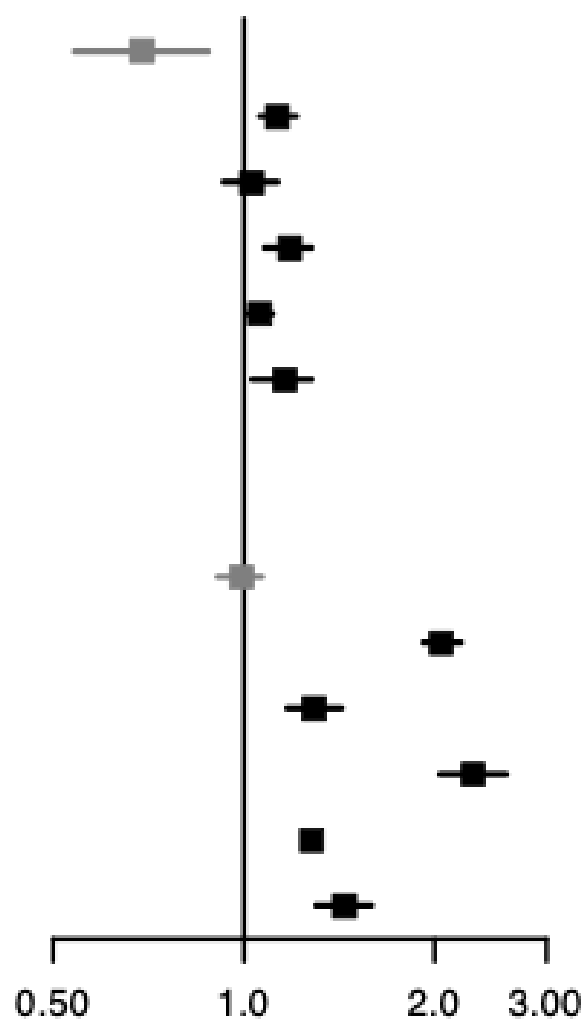

Comment 5: The authors constructed "monthly hypothetical trials." It is unclear how they handled the correlation of data for patients who were eligible in multiple consecutive months. If the same patient appears in multiple "trials" without appropriate variance adjustment, such as robust sandwich estimators or bootstrap methods, the confidence intervals may be artificially narrow.

Thank you for raising this important methodological point. To address this concern, we re-estimated all 95% confidence intervals using cluster-robust (sandwich) variance estimators, with clustering at the patient level. This approach accounts for within-patient correlation arising from repeated enrolment across monthly trial emulations. All reported results and corresponding figures have been updated accordingly.
We have clarified this explicitly in the Methods section as follows:

"95% confidence intervals were estimated using cluster-robust (sandwich) variance estimators grouped at the patient level to account for intra-individual correlation."

Reviewer #3 (Remarks to the Author):

I have read with great interest the manuscript entitled " Evaluating bias in target trial emulation of heart failure therapies: a comparison of classical and deep learning adjustment methods" submitted to the Nature Communications. This original study selected patients with heart failure with reduced ejection fraction and emulated target trials initiated monthly from January 1, 24 2010, to January 1, 2015, to assess the effects of β blockers (positive control) and digoxin 25 (negative control) versus usual care on two-year all-cause mortality. They applied four adjustment strategies: propensity score matching (PSM), inverse probability of treatment weighting, targeted maximum likelihood estimation, and a transformer based deep learning approach, benchmarking estimates against RCT findings. The authors should be congratulated for performing such a novel and comparative analysis. While the manuscript is well-structured and insightful, it has several methodological, reporting, and interpretive limitations, that should be acknowledged.

1. Please the authors to add a reference in the first paragraph of Introduction.

Thank you for pointing this out. We have added an appropriate reference in the first paragraph of the Introduction.

2. While the authors recognize the limitations of TTE, they have not emphasized on the positive aspects of this approach. It should be clarified in the second paragraph of Introduction.

We agree and have revised the second paragraph of the Introduction as follows:

"This approach involves specifying a hypothetical trial protocol with eligibility criteria, treatment strategies, outcomes, and follow-up periods. By aligning the start of follow-up ('time zero') with the point at which eligibility is met and treatment assignment is determined, the framework establishes a common baseline across comparison groups and mitigates design-related biases*, such as immortal time bias."*

3. Line 93: How is usual care defined?

As our study used CPRD Aurum, a UK primary care-based database, we defined "usual care" as routine management provided in primary care. In the UK healthcare system, patients with chronic conditions who are actively registered with a general practitioner (GP) typically receive their ongoing clinical management through primary care services.

4. Lines 95-97: Digoxin has not shown benefit in patients with HFrEF, but neither harm. Could it be included as negative control?

a. Recently, a RCT showed that digitoxin led to a lower combined risk of death from any cause or hospital admission for worsening heart failure than placebo among patients with heart failure and reduced ejection fraction who received guideline-directed medical

therapy. (10.1056/NEJMoa2415471). How might this affect the selection of digoxin as negative control.

Thank you for this important suggestion. We agree that the recent DIGIT-HF trial is relevant and warrants discussion. However, although digitoxin demonstrated a reduction in the composite endpoint of death or first hospital admission for worsening HF, it did not show a clear reduction in all-cause mortality. Therefore, its findings remain consistent with our use of digoxin as a negative control for all-cause mortality.

We have now added the following paragraph to the Discussion:

"Digoxin was selected as a negative control on the basis of prior randomised trial evidence indicating no effect on all-cause mortality. More recently, the DIGIT-HF trial evaluated digitoxin, a closely related cardiac glycoside, compared with placebo. The trial reported a reduction in the composite endpoint of death or first hospitalisation for worsening HF, but no clear reduction in all-cause mortality (HR 0.86, 95% CI 0.69–1.07). Taken together, these findings suggest that cardiac glycosides may reduce HF morbidity without clear evidence of harm with respect to mortality. In contrast, our observational analyses consistently yielded elevated mortality estimates for digoxin. The divergence between contemporary trial findings and routine-care estimates reinforces the importance of randomised evidence in this setting and highlights that, even with advanced adjustment methods and comprehensive longitudinal data, deriving reliable treatment effect estimates remains challenging in complex HF populations."

5. Have the authors pre-registered the protocol of their study? If yes, it should be acknowledged.

This study was not prospectively pre-registered. However, the protocol received scientific approval from the CPRD Independent Scientific Advisory Committee (protocol number: 20_095). As stated in the Methods section:

"The CPRD Group holds ethical approval from the National Research Ethics Service Committee for all observational studies conducted using CPRD. This study received scientific approval from the CPRD Independent Scientific Advisory Committee (protocol number: 20_095)."

6. Line 125: Why the patients should be registered with a GP for at least two years before enrolment?

Thank you for this comment. We have now changed this to one year. Since we applied a one-year washout period and extracted baseline covariates at trial enrolment, requiring at least one year of continuous registration ensures that patients have sufficient medical history recorded to support confounding adjustment.

We have clarified this in the Methods section as follows:

"To ensure sufficient baseline information for covariate assessment and to exclude prevalent users, we required at least one year of continuous registration with a general practice before enrolment."

7. Why did the authors select a single outcome of their analysis?

The primary endpoints of the two benchmark trials were all-cause mortality, unlike more recent trials that have used cardiovascular mortality as the primary outcome. To maintain consistency with the original trial designs, we focused on replicating the primary endpoint. As we were unable to replicate the primary RCT outcomes, we believe that reporting secondary endpoints would provide limited additional insight and could potentially be misleading. We also attempted to replicate heart failure hospitalisation benefits but were similarly unable to reproduce the trial findings.

8. Lines 157, 171, 184. The abbreviations should be avoided in the start of each sentence.

Thank you for noting this. We have revised to spell out the full terms at the beginning of sentences.

9. Table 1.
a. Dapaglifozin and Entresto should have been included in the Medications, considering that both are first-line treatment for HFrEF.
I suspect that there are not many in the dataset?
b. Table 1. There are no benign malignancies.
c. Except systolic and diastolic BP, I would also HR as it plays important role in the prognosis of such patients.

Our study period was 2010–2015. During this time, SGLT2 inhibitors were not guideline-recommended therapies for heart failure, and sacubitril/valsartan (Entresto) had not yet been widely adopted in UK clinical practice. We confirmed that fewer than 100 patients in our cohort were receiving SGLT2 inhibitors during the study period. Given their very low prevalence, inclusion of these therapies as baseline covariates would not meaningfully affect confounding adjustment.

We have now additionally included eGFR, heart rate, HDL cholesterol, total cholesterol, haematological cancer, and solid cancer as covariates, re-run the analyses, and updated the results. Heart rate is indeed an important variable for propensity score estimation, and our conclusions remained unchanged; we were still unable to replicate the RCT effects using any method (results attached below).

Method	Total number of participants	Initiators (n/N)	Non-Initiators (n/N)	Effect Estimate (95% CI)
**Beta-blockers**				
RCT	3,039	134 / 1,723	159 / 1,316	0.69 (0.54–0.88)
Crude	417,330	994 / 8,076	45,866 / 409,254	1.13 (1.06 - 1.21)
PSM	16,152	994 / 8,076	989 / 8,076	1.03 (0.93 - 1.13)
IPTW	413,158	976 / 7,995	45,287 / 405,163	1.18 (1.08 - 1.28)
TMLE	413,158	976 / 7,995	45,287 / 405,163	1.06 (1.02 - 1.11)
DL	167,691	987 / 7,985	17,687 / 159,706	1.16 (1.03 - 1.28)
**Digoxin**				
RCT	6,800	1,181 / 3,397	1,194 / 3,403	0.99 (0.91–1.07)
Crude	1,678,203	907 / 4,377	186,425 / 1,673,826	2.05 (1.92 - 2.20)
PSM	8,754	907 / 4,377	747 / 4,377	1.29 (1.17 - 1.43)
IPTW	1,661,420	899 / 4,333	183,018 / 1,657,087	2.30 (2.04 - 2.60)
TMLE	1,661,420	899 / 4,333	183,018 / 1,657,087	1.28 (1.25 - 1.30)
DL	90,852	900 / 4,326	9,531 / 86,526	1.44 (1.30 - 1.59)

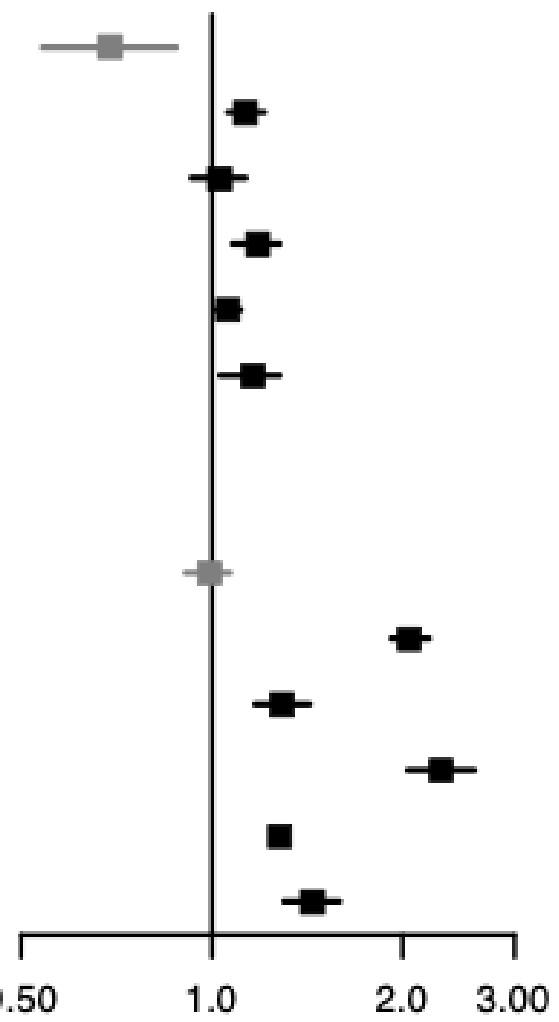

10. Lines 252-253: Does the adherence in beta-blockers and digoxin could affect the results?

We consider adherence in both cohorts to be high, as reflected by the proportion of days covered: beta-blockers (median 85.5%, IQR 55.9–93.7%) and digoxin (median 83.0%, IQR 45.8–93.7%). It is also unlikely that the substantially elevated mortality estimates observed for digoxin can be explained by adherence.

11. Table 2: Valve disorders were not selected in the prespecified variables, as presented in Table 1.

Thank you for noting this. Valve disorders were included as adjustment covariates in our models but were inadvertently omitted from the original version of Table 1 listing the prespecified variables. To avoid redundancy and potential confusion, we have now removed the original covariate list table, as the demographic table already presents all included covariates.

12. As presented in Figure 2, discrepancies in beta-blockers are smaller compared to Digoxin. How is this explained?

We believe this difference reflects variation in the degree of confounding by indication. Beta-blockers are guideline-recommended first-line therapies for HFrEF and are more broadly prescribed across eligible patients. In contrast, digoxin is typically reserved for patients with more advanced disease or specific clinical indications, which may be associated with a higher underlying risk profile. As a result, treatment allocation for digoxin is more strongly influenced by disease severity, leading to greater confounding and larger discrepancies between observational estimates and RCT findings.

13. The authors should add in the Discussion how their study in the current research practice and which method to be selected.

As none of the methods were able to recover the RCT estimates in our study, we suggest that in settings with substantial confounding, greater reliance should be placed on randomised evidence in the Discussion:

"Where clinical stakes are high, decisions should prioritise randomised evidence; where trials are infeasible, triangulation across multiple observational designs, the use of negative and positive controls, and extensive, transparent sensitivity analyses are essential for credible inference."

Reviewer #4 (Remarks to the Author):

I co-reviewed this manuscript with one of the reviewers who provided the listed reports. This is part of the Nature Communications initiative to facilitate training in peer review and to provide appropriate recognition for Early Career Researchers who co-review manuscripts.

Reviewer #1 (Remarks to the Author):

The answers were well-drafted although finally there is only a minor improvement. New drugs were not included in the analysis. The methodology is good but the clinical significance remains limited for practice.

We thank the reviewer for this comment. As an evaluation study using observational data, our work is intended primarily to inform the interpretation of observational evidence rather than current HF clinical management, which remains guided by randomised trial evidence. In this regard, we believe the study has important implications for future policy decision-making. As observational evidence derived under the TTE framework is receiving increasing attention, and in some settings considerable confidence from regulators and policy makers, our findings highlight the need for caution in placing strong confidence in observational estimates.

Reviewer #2 (Remarks to the Author):

The authors have addressed the previous comments appropriately, and I have no further comments.

Reviewer #3 (Remarks to the Author):

I thank the authors for their thorough and detailed response to the reviewers' comments. The revision is substantive and demonstrates genuine engagement with the concerns raised. The manuscript has improved considerably, and I am broadly satisfied that the major concerns have been addressed. However, several residual issues require attention before further evaluation. More specifically;

1. Reviewer 1 asked the authors to explore subgroups to detect treatment effect modification, "including interactions in the model." The authors responded with AF-stratified analyses only. While the AF stratification is clinically relevant and well-presented, the response does not acknowledge the narrowness of this choice. In a study of this scale, with >400,000 participants and 39 baseline covariates, the absence of any exploration of other clinically relevant effect modifiers (e.g., age, sex, renal function, baseline heart rate, diabetes status) is notable. I recognise that the primary emulation failed to replicate the main RCT effect, which limits the interpretability of subgroup findings. However, this reasoning should be stated explicitly in the reviewer's response. A brief statement in the Discussion acknowledging that broader subgroup analyses were not performed and explaining why would be appropriate.

We thank the reviewer for raising this point. As you noted**,** observational studies generally benefit from investigating clinically relevant subgroups, and the rationale for not doing so in this study should be made explicit to help readers understand. We have therefore added the following statement to the limitations section:

"Fourth, we did not conduct extensive subgroup analyses, because the primary emulations were not consistent with the benchmark RCT findings, which limited the value of further subgroup analyses."

2. The response provides a reasonable definition of "usual care" as routine primary care management. However, this definition does not appear to have been incorporated into the manuscript itself. The Methods section and Results section refer to "usual care" without explicit definition. Since readers of the published manuscript will not have access to the rebuttal, this definition should be added to the Methods.

We agree that this should be clarified to avoid confusion. We have now added the following text to the Methods:

"In this study, usual care referred to routine clinical management provided through UK primary care. As all participants were actively registered with a general practice, those in the control group were assumed to continue receiving ongoing management for HFrEF through primary care services."

3. The authors commendably re-extracted the beta-blocker cohort with 40–80 years to match the benchmark RCT. However, the Methods (line 395–396) still state that the digoxin cohort uses an age range of 35–85 years. The DIG trial enrolled patients aged 21 and over. The rebuttal does not explicitly clarify whether Reviewer 2's concern applied only to the beta-blocker cohort or to both cohorts. For transparency, the authors should add a brief justification for the 35–85 age restriction in the digoxin emulation, explaining how it relates to the DIG trial's eligibility criteria and whether this narrower range might itself contribute to discordance with the original trial estimates.

We thank the reviewer for this important point. For the digoxin emulation, we applied an age range of 35–85 years to better reflect the age distribution of the DIG trial. The DIG trial enrolled adults aged 21 years and older, but the published age distribution shows that participants older than 80 years were relatively uncommon (5.5%). In routine care, the HF population includes a larger proportion of very elderly patients, particularly those over 85 years. We initially attempted to apply the full DIG eligibility (≥21 years), but this resulted in a mean age substantially higher than the mean reported in the DIG trial. To maintain comparability with the trial population, we therefore applied the 35–85 year age restriction. We have clarified this in the Methods:

"For the digoxin emulation, although the original DIG trial enrolled adults aged 21 years and older, only a small proportion of participants were older than 80 years.[33] Because routine care includes a greater proportion of very elderly patients with HF than the trial population, directly applying the original age criterion would have produced a less comparable age distribution. We therefore applied an age restriction of 35–85 years for the digoxin emulation."

4. The Discussion briefly mentions that active-comparator designs "may help alleviate some of these imbalances" (lines 319–321). Given the central finding that confounding by indication drives the discordance, particularly for digoxin, I would encourage the authors to expand this point. Specifically, how might the study conclusions differ if the authors had employed an active-comparator new-user design (e.g., beta-blocker vs. ACE inhibitor initiation, or digoxin vs. another rate-control agent)?

We agree and have expanded the limitations section as follows:

"Third, we benchmarked against placebo-controlled trials only. Such emulations are especially vulnerable to confounding by indication and may be less robust than active-comparator designs, which compare patients initiating alternative therapies at a more similar point in the clinical decision process."

However, we cannot determine whether use of an active-comparator design would have materially changed our conclusions. As also noted in the second paragraph of the Discussion, the RCT-DUPLICATE initiative, which used PSM within an active-comparator TTE framework, reproduced RCT outcomes in only 6 of 10 emulations, suggesting that active-comparator designs do not necessarily eliminate this confounding problem.

Reviewer #4 (Remarks to the Author):

I co-reviewed this manuscript with one of the reviewers who provided the listed reports. This is part of the Nature Communications initiative to facilitate training in peer review and to provide appropriate recognition for Early Career Researchers who co-review manuscripts.
